# Completion of BAX recruitment correlates with mitochondrial fission during apoptosis

**DOI:** 10.1038/s41598-019-53049-w

**Published:** 2019-11-12

**Authors:** M. E. Maes, J. A. Grosser, R. L. Fehrman, C. L. Schlamp, R. W. Nickells

**Affiliations:** 1 Department of Ophthalmology and Visual Sciences, University of Wisconsin – Madison, Madison, WI USA; 2 Institute of Science and Technology Austria, Klosterneuburg, Austria

**Keywords:** Tumour-suppressor proteins, Apoptosis

## Abstract

BAX, a member of the *BCL2* gene family, controls the committed step of the intrinsic apoptotic program. Mitochondrial fragmentation is a commonly observed feature of apoptosis, which occurs through the process of mitochondrial fission. BAX has consistently been associated with mitochondrial fission, yet how BAX participates in the process of mitochondrial fragmentation during apoptosis remains to be tested. Time-lapse imaging of BAX recruitment and mitochondrial fragmentation demonstrates that rapid mitochondrial fragmentation during apoptosis occurs after the complete recruitment of BAX to the mitochondrial outer membrane (MOM). The requirement of a fully functioning BAX protein for the fission process was demonstrated further in *BAX*/*BAK*-deficient HCT116 cells expressing a P168A mutant of BAX. The mutant performed fusion to restore the mitochondrial network. but was not demonstrably recruited to the MOM after apoptosis induction. Under these conditions, mitochondrial fragmentation was blocked. Additionally, we show that loss of the fission protein, dynamin-like protein 1 (DRP1), does not temporally affect the initiation time or rate of BAX recruitment, but does reduce the final level of BAX recruited to the MOM during the late phase of BAX recruitment. These correlative observations suggest a model where late-stage BAX oligomers play a functional part of the mitochondrial fragmentation machinery in apoptotic cells.

## Introduction

The *BCL2* gene family works cooperatively to initiate and execute the intrinsic apoptotic pathway. BAX, a pro-apoptotic family member, is responsible for executing the committed step of the intrinsic apoptotic program^1^. BAX predominantly resides in the cytosol as an inactive monomer, but once activated, undergoes a conformational change^2^ that catalyzes dimerization at the mitochondrial outer membrane (MOM)^3,4^, the site of recruitment^5,6^, leading to oligomer formation and MOM permeabilization (MOMP)^7,8^. BAX oligomer formation and MOMP facilitate the release of cytochrome c^9,10^, and are often considered the ‘point of no return’ in the apoptotic program^11^.

One hallmark of apoptosis is mitochondrial fragmentation, which is the product of halted mitochondrial fusion and increased mitochondrial fission^12–14^. Mitochondria are dynamic organelles constantly undergoing fission and fusion, the process of mitochondrial separation and reconnection, which occurs asynchronously within a cell at steady state. The rates of fission and fusion are balanced during the steady-state in order to create an equilibrium necessary to each cell’s needs^15^. Mitochondrial fission is primarily regulated by DRP1^16^, dynamin 2 (DYN2)^17^, with the aid of adaptor proteins Fis1^18^, and mitochondrial dynamics proteins 49 and 51 (MiD49/51)^19^. DRP1 is a dynamin-like large GTPase which, like dynamin, will lasso around and constrict mitochondria at the point of scission^20^. Mitochondrial fusion requires separate machinery including OPA1^21^, MFN1 and MFN2^22^. Surprisingly, BAX and its homologue BAK, are also essential components of the fusion process in healthy cells and are thought to help localize MFNs to sites of fusion through direct binding^23–25^. During apoptosis, the dynamic mitochondrial equilibrium is disrupted as fission predominates and fusion is halted, resulting in widespread mitochondrial fragmentation. Importantly, the switch to apoptosis is controlled by BAX, preventing it from participating in fusion^14^. Steady-state fission and apoptosis-associated fission appear to be governed by separable processes, which could then be regulated by different mechanisms^26^.

The process of BAX recruitment occurs rapidly once initiated, and follows a sigmoid pattern of recruitment, which exhibits an early and late phase^27,28^. The early phase, or initiation of BAX recruitment, includes dimer and oligomer formation at the MOM, leading to MOMP and apoptotic molecule release^9,10^. The late phase, or the completion of BAX recruitment, is the point at which the exponential growth of the BAX curve is suspended and reaches a plateau. It has been assumed that the function of the BAX oligomer is to release cytochrome c and initiate the downstream caspase cascade, but since the release of cytochrome c and other pro-apoptotic signaling molecules occurs during the early recruitment phase^28^, this provides no explanation for its continued recruitment to the MOM, or for the pivotal plateau commencement where BAX recruitment concludes.

One potential function for the completion of BAX recruitment is regulation of mitochondrial fission. Although BAX and the mitochondria are consistently associated, the direct relationship between mitochondrial fragmentation and apoptotic machinery has not been fully described^12,29,30^. After an apoptotic stimulus, BAX clusters at the cardiolipin-rich mitochondrial scission sites^16^, where it colocalizes with DRP1^31^. Mitochondrial scission creates a change in membrane curvature, which has been shown to facilitate integration of membrane-associated proteins^32,33^, and studies have suggested that DRP1 may stimulate BAX oligomerization^34^. While these data are critical to outline the setting for BAX and fission during apoptosis, it is possible that the recruitment of BAX is an epiphenomenon associated with membrane curvature, DRP1 localization, cardiolipin enrichment at the scission sites, or some combination of all these features. Alternatively, other studies have indicated that overexpression of BAX can enhance mitochondrial fragmentation after induction of apoptosis, or even spontaneously initiate fragmentation in the absence of an inducer^12^. These latter observations, along with the localization studies showing BAX aggregations at scission sites, has strongly implicated the existence of a BAX-dependent role in mitochondrial fragmentation.

Temporally, it has been shown that mitochondrial fragmentation occurs after cytochrome c release, which is associated with initial BAX recruitment^35^, suggesting that MOMP may be mechanistically independent of fission during apoptosis^30^. In studies using the protozoan *Trypanosoma brucei*, an organism with a single mitochondrion and no apoptotic machinery, expression of exogenous BAX induces cytochrome c release, loss of MOM potential, and mitochondrial fission, in separable and sequential order^36^. Here, we show that completion of BAX recruitment is temporally associated with a rapid loss of mitochondrial volume, and that cells expressing a BAX mutant retaining its fusion properties but with impaired ability to translocate to the MOM during apoptosis, fail to exhibit apoptosis-induced mitochondrial fragmentation. Altering DRP1 function in apoptotic cells has no effect on the initiation or rate of BAX recruitment, but does modulate the total level of BAX that is recruited in the plateau phase of the process.

## Results and Discussion

### Completion of BAX recruitment coincides with a period of rapid mitochondrial volume decrease

**Figure 1:**
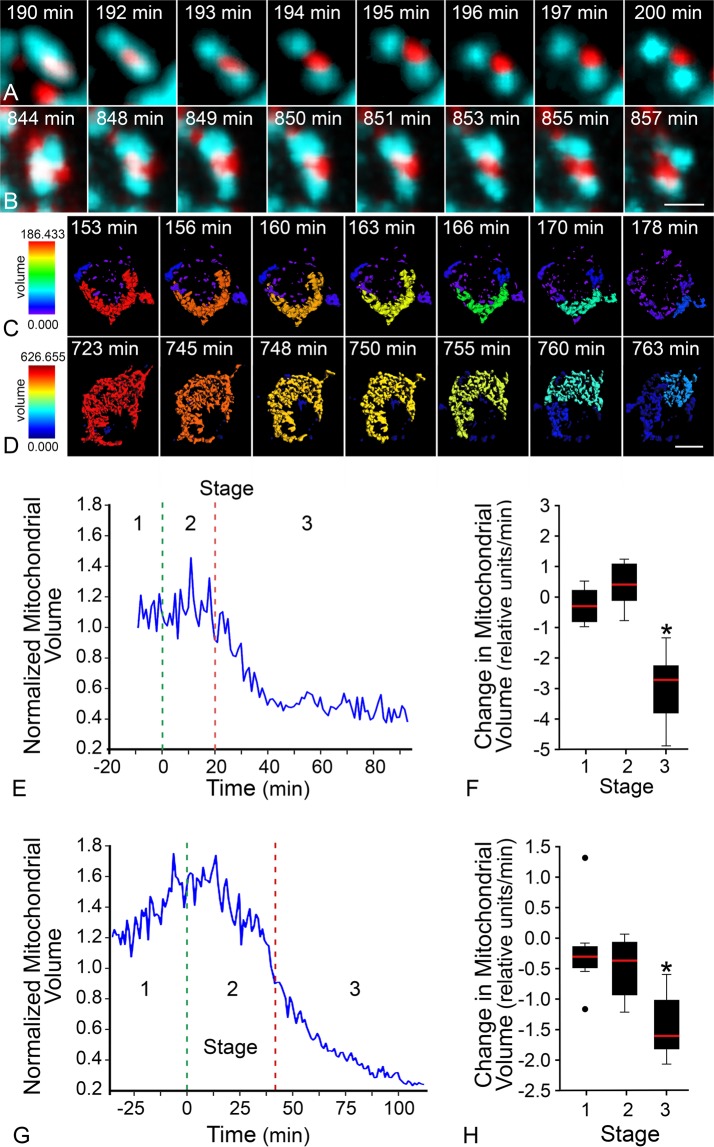
BAX recruitment completion coincides with rapid decline in mitochondrial volume. Mitochondrial fission occurs at the site of BAX recruitment. Images from a time-lapse video show recruited wild type mCherry-BAX at isolated mitochondria (mito-BFP, pseudo-colored teal) as the fission process completes in (**A**) a D407 cell and (**B**) a 661W cell. In these time series, the first and last image depicts a representative timepoint before and after fission, while the remaining images demonstrate the consecutive timeframes as the mitochondria are separating. Mitochondrial volume is graphically represented as a heat map of a 3-dimensional reconstruction of contiguous mitochondrial surfaces identified in a series of 20–25 optical sections. Red corresponds to reconstructions with the largest volume, and blue to the smallest. Images from time-lapse videos of color-coded mitochondria indicate volume changes at times after (**C**) staurosporine treatment in a D407 cell or (**D**) forced HDAC3 expression in a differentiated 661W cell (see also Video 1). Note that D407 cells have substantially smaller mitochondria than 661W cells prior to BAX recruitment. Representative graphs show the average (normalized) volume of mitochondria after an apoptotic stimulus within (**E**) a D407 cell or (**G**) a 661W cell. Each graph also depicts the average time of BAX recruitment initiation (green line) and BAX recruitment completion (red line) for that specific cell. The time between each of these distinctions creates three stages; Stages 1, 2, and 3, identifying 20 minutes before, during and 20 minutes after BAX recruitment. (**F**) D407 cells showed a Stage 1 rate of change of −0.29 ± 0.6 relative mitochondrial volume (RMV)/min, Stage 2 rate of 0.73 ± 1.2 RMV/min, and Stage 3 rate of −3.0 ± 1.2 RMV/min (n = 6 cells), (**H**) while 661W cells showed Stage 1 rate of change of −0.19 ± 0.8 RMV/min, Stage 2 rate of −0.50 ± 0.5 RMV/min, and Stage 3 rate of −1.45 ± 0.5 RMV/min (n = 4 cells). For both cell types, the rate of change of mitochondrial volume in Stage 2 was statistically similar to Stage 1 by a two-tailed *t*-test (D407 p = 0.28, 661W p = 0.45). The rate of change in Stage 3 was significantly faster than Stage 1 (D407, 661W *p < 0.05). Size bar = 500 nm (**A**,**B**), 3 µm (**C**,**D**)

BAX recruitment to the MOM was assessed by time-lapse imaging of live cells expressing fluorescently tagged BAX. Once initiated, the recruitment process was complete within 10–20 minutes. Kinetic analysis at individual mitochondrial foci showed that recruitment followed a sigmoid curve in all cell types examined, which allowed us to mathematically define the times for initiation and completion of the recruitment process (Fig. S1). Detailed methods for quantitation of BAX recruitment can be referenced in Maes *et al*.^28^. Mitochondria were identified using a mito-BFP tag and changes in these organelles were monitored and quantitated in a temporal series of Z-stacked images. Three-dimensional objects were rendered from these images using IMARIS 7.7 software, which determined the volume of contiguous organelles. For each cell, the average mitochondrial volume of all objects was calculated for the duration of the time-lapse images and converted to a heat map. Figure 1 shows a comparison of wild type BAX recruitment and mitochondrial changes in two different cell types undergoing apoptosis. Recruited BAX formed characteristic puncta that accumulated at the fission sites of fragmenting mitochondria (Fig. 1A,B) as previously described^31,37^. Heat map representations of mitochondrial volume showed that both cell types exhibited a dramatic decrease in the average volume of all mitochondrial objects as they underwent apoptosis (Fig. 1C,D, Video 1). Data from individual cells were evaluated for the change in average mitochondrial volume during the 20 minute interval before the initiation of BAX recruitment (Stage 1), during recruitment (Stage 2), and 20 minutes after completion of BAX recruitment (Stage 3) (Figs 1E,G, S1). Box and whisker plots show the rate of average volume change for each stage. For Stages 1, 2, and 3, respectively, D407 cells showed rates of −0.29 ± 0.6 relative mitochondrial volume per minute (RMV/min), 0.73 ± 1.2 RMV/min, and −3.0 ± 1.2 RMV/min (Fig. 1F), while 661W cells showed rates of −0.19 ± 0.8 RMV/min, −0.50 ± 0.5 RMV/min, and −1.45 ± 0.5 RMV/min (Fig. 1H). In a separate experiment, the change in mitochondrial volume was assessed in human ARPE-19 cells treated with staurosporine (STS). BAX recruitment initiation and termination were determined manually in these cells, so these data were not included in Fig. 1. However, a similar pattern of mitochondrial volume change was observed in these cells as well (Fig. S2). All cell types showed a significant decrease in average mitochondrial volume after completion of BAX recruitment (Stage 3) when compared to Stage 1 as baseline (p < 0.05). Notably, there was no significant difference between Stage 1 and 2 rates in each cell type (D407 p = 0.28, 661W p = 0.45, ARPE-19 p = 0.06). These data show that completion of BAX recruitment identifies the point of rapid mitochondrial volume changes within an apoptotic cell.

MOMP and fission have previously been shown to be separate events both temporally^35,38^ and mechanistically^30^. In MOMP, cytochrome c may be released, theoretically, through a pore induced by a single BAX molecule^39^, or more likely from a single BAX dimer^8^. Kinetic analysis of mCherry-BAX recruitment and cytochrome c-GFP or SMAC/Diablo-GFP release in D407 cells indicates that MOMP is rapidly induced and essentially complete very early in the process of BAX recruitment and well in advance of mitochondrial fragmentation in these cells^28^. Others have demonstrated that BAX monomers are rapidly integrated into dimers and then oligomers once the process of recruitment is initiated^8,40^. While continued growth of the oligomer may function to increase pore size, allowing for the release of larger molecules^8,40^, the correlation between the end of BAX recruitment and the onset of mitochondrial fission further supports a mechanistic role of the BAX oligomer in the fission process.

### BAX is essential for apoptosis-induced mitochondrial fission

The requirement for BAX in mitochondrial fission has been inferred by its localization to scission sites, its relationship with DRP1, and observations that overexpression of exogenous BAX can lead to spontaneous fragmentation^12,29^. The ability to accelerate the conversion of elongated to punctiform mitochondria during apoptosis^16^, however, has not been directly tested in cells lacking pro-apoptotic proteins. A principal complication hindering this analysis is that cells lacking both BAK and BAX have a more fragmented network of mitochondria compared to wild type counterparts due to a lack of fusion^14,25,41^. Consequently, after induction of apoptosis, there is no further decrease in mitochondrial size. Therefore, we implemented a system using HCT116^*BAX*−/−*/BAK*−/−^ double knockout cells expressing either a wild type or a mutant BAX construct. The mutant BAX (P168A mCherry-BAX) has a proline substituted for alanine at amino acid 168 located at the junction between α8 and α9 (transmembrane domain) helices, and lacks the ability to fully recruit to the MOM^42^.

**Figure 2:**
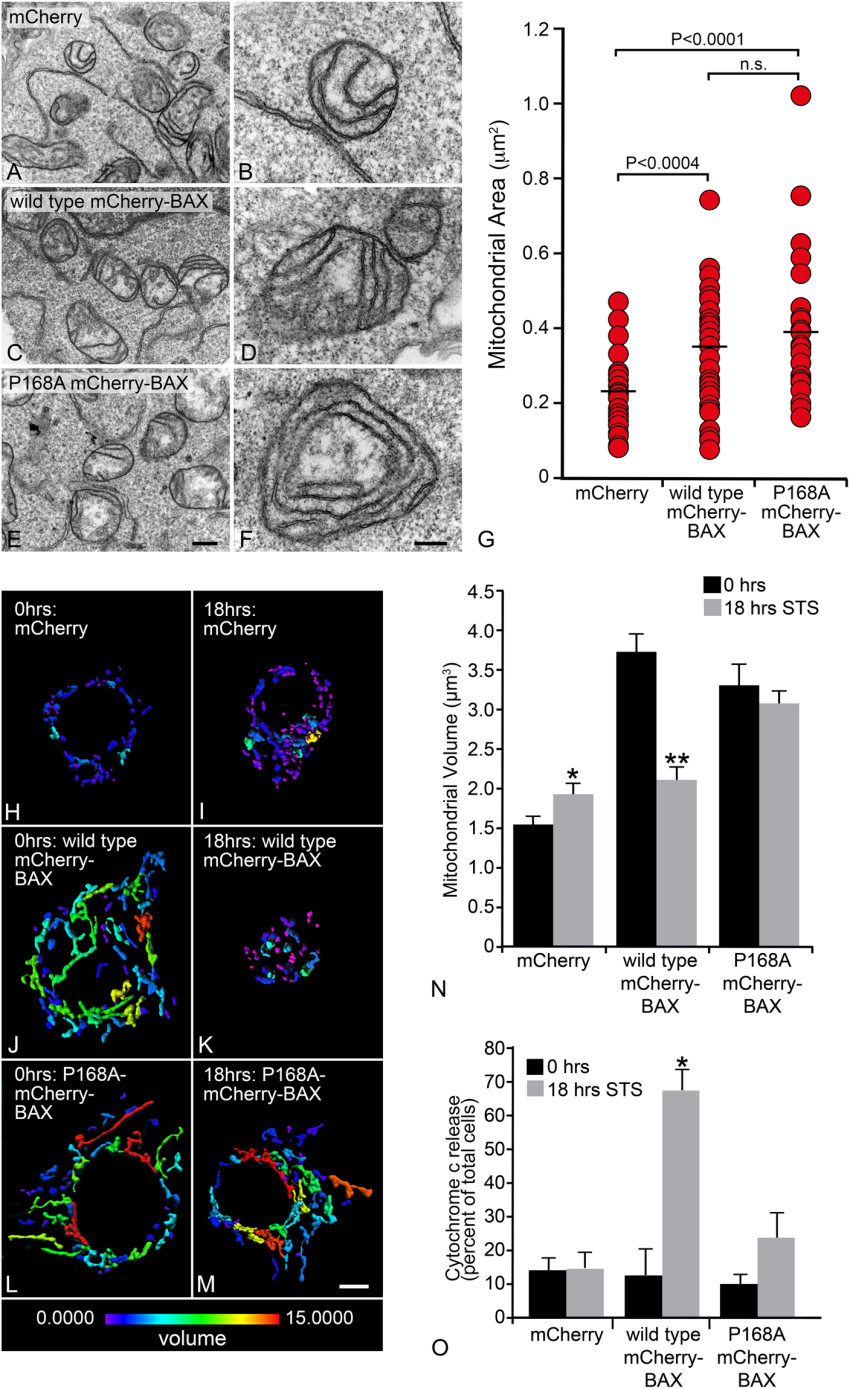
The apoptotic function of BAX is required for mitochondrial fragmentation. Representative transmission electron microscope images of HCT116^*BAX*−/−*/BAK*−/−^ cells expressing (**A**,**B**) mCherry, (**C**,**D**) wild type mCherry-BAX, or (E, F) P168A mCherry-BAX. All conditions exhibit intact double membranes and tubular cristae formation. (**G**) Mitochondrial area was calculated using ImageJ (v1.42q) on mitochondria exhibiting a circular cross section to eliminate sectioning bias. The average area for mCherry was 0.218 ± 0.016 (±s.e.m.), wild type mCherry-BAX was 0.327 ± 0.025, and P168A mCherry-BAX was 0.385 ± 0.030 µm^2^ (n ≥ 35 mitochondria per condition). Both wild type and P168A mCherry-BAX exhibited similar mitochondrial area (p = 0.134), which were each significantly greater when compared to mCherry alone (p < 0.004 and p < 0.0001, respectively). (A, C, E) Size bar = 1 µm. (**B**,**D**,**F**) Size bar = 200 nm. (**H**–**M**) Heat maps of mitochondrial volume are shown for representative cells, before and 18 hrs after staurosporine (STS) addition. A numerical based color-coded scale from 0 µm^3^ to 15 µm^3^ is shown at the bottom of the panels. Panels H and I show mCherry only control at 0 h and 18 hr after STS. Both conditions contain fragmented mitochondria and are primarily colored on the blue end of the volume spectrum. Panels J and K show wild type mCherry-BAX at 0 h with elongated mitochondria in the green to red spectrum, signifying larger volumes. With STS treatment, wild type mCherry-BAX shows mitochondrial fragmentation, exhibited by primarily blue colored objects. Panels L and M show P168A mCherry-BAX, which exhibits elongated mitochondria in the green to red spectrum in both conditions, demonstrating that P168A can rescue mitochondrial fusion in these cells (compare to **H**), but the process of mitochondrial fragmentation is impaired. Size bar = 5 µm. (**N**) A graph showing the mean mitochondrial volumes for each condition from three separate experiments (n = 30 cells per condition). For each cell analyzed, the average volume of all mitochondrial objects was used. The average volumes for mitochondria for each condition are as follows: mCherry alone (0 h) 1.6 ± 0.1 µm^3^ (±s.e.m.) and (18 h) 1.9 ± 0.1 µm^3^ (*p < 0.05), wild type mCherry-BAX (0 h) 3.7 ± 0.2 µm^3^ and (18 h) 2.1 ± 0.1 µm^3^ (**p < 1 × 10^−7^), and P168A mCherry-BAX (0 h) 3.3 ± 0.3 µm^3^ and (18 h) 3.1 ± 0.1 µm^3^ (p = 0.44). For mCherry (18 h) and wild type mCherry-BAX (18 h), (p = 0.36). For wild type mCherry-BAX (0 h) and P168A mCherry-BAX (0 h), (p = 0.23). The P168A BAX mutant also impairs cytochrome c release after STS addition. (**O**) A graph shows the percentage of total cells exhibiting the release of cytochrome c-GFP (based on diffuse localization in the cytoplasm, see supplemental Fig. S5). Each condition exhibits at least 85% of cells with cytochrome c-GFP localized to the mitochondria, except in wild type mCherry-BAX at 18 hours after STS addition, where the majority of cells (68%) show cytoplasmic localization of cytochrome c-GFP. There is no statistical difference between the percentage of cells that exhibit cytoplasmic cytochrome c-GFP localization at 0 and 18 hours in the mCherry condition (p = 0.93) or in the P168A mCherry-BAX condition (p = 0.06). There was a significant difference in the numbers of cells with cytoplasmic cytochrome c-GFP in the wild type mCherry-BAX condition after STS addition (*p < 0.0005). The total number of cells was counted from three separate experiments (n ≥ 125 cells per condition, except 18 hours wild type mCherry-BAX where n = 87 cells).

HCT116^*BAX*−/−*/BAK*−/−^ cells were nucleofected with 1 µg mito-BFP and 1 µg of either mCherry, wild type mCherry-BAX or P168A mCherry-BAX, and 500,000 cells were plated on coverslip bottomed 35 mm tissue culture dishes. Cells were grown for three days to allow for mitochondrial dynamics to equilibrate prior to apoptosis induction using 1 µM STS. HCT116^*BAX*−/−*/BAK*−/−^ cells expressing wild type or P168A mCherry-BAX showed elongated mitochondria three days after nucleofection, while cells expressing empty mCherry (control) exhibited fragmented mitochondria (Fig. S3). Mitochondrial morphology defects in cells lacking BAX and BAK, along with recovery of morphology after wild type BAX expression, are consistent with previous reports^25^. Electron microscopy of mitochondria in cells expressing the same three constructs confirmed that the mitochondrial cristae structure and integrity were intact, showing no differences between wild type and P168A BAX, or between cells expressing BAX constructs and control cells expressing only mCherry (Fig. 2A–F). Both wild type and P168A mCherry-BAX expressing cells exhibited similarly sized mitochondria, which were significantly greater in cross-sectional area when compared to mCherry alone (Fig. 2G, p < 0.0004).

These results suggest that conformational changes in the C-terminus of BAX are not critical for a functional role in fusion. This aligns with previous results from Hoppins and colleagues^23^ demonstrating that only the soluble form of BAX participates in fusion. Both activated BAX and a tethered non-oligomerizing mutant (BAX 1-2/L-6) fail to participate in fusion, suggesting that BAX rearranges to interact with MFN2 for fusion at the MOM. Since P168A BAX successfully executes fusion, it must also be shuttled to the MOM^43^, and interact with MFN2 to achieve this^44,45^.

After mitochondrial recovery, cells were challenged with STS to induce apoptosis. At 18 hours after induction, no changes in mitochondrial morphology were present in the mCherry only (control) condition, which remained predominantly small and fragmented (Fig. S3B). In the presence of wild type mCherry-BAX, apoptosis induction caused a shift from mitochondria exhibiting a connected network (Fig. S3A), to small and fragmented organelles (Fig. S3B). This was correlated with the recruitment of wild type mCherry-BAX to form puncta at the mitochondria. Importantly, in the presence of P168A mCherry-BAX, apoptosis induction did not change the mitochondrial morphology, as the mitochondria remained filamentous and connected, and P168A mCherry-BAX remained cytoplasmic (Fig. S3B).

To quantify the morphological differences observed between each condition, mitochondrial volume measurements were calculated for all mitochondria within a cell. IMARIS 7.7 was used to map, in three dimensions, the mitochondria labeled with mito-BFP. In Fig. 2H–M, heatmap representations of the volume of mitochondria within a representative cell for each condition are shown. The average volume of all mitochondria within a cell was calculated. Therefore, a cell with fragmented mitochondria would be represented by a small average volume per object. From three separate experiments, the average mitochondrial volume for cells expressing wild type mCherry-BAX before and after STS addition was 3.7 ± 0.2 µm^3^ (±s.e.m.) and 2.1 ± 0.1 µm^3^ (Fig. 2N), respectively, showing a statistically significant change (p < 1 × 10^−7^). These two measurements provide the best representation for a filamentous network of mitochondria in a healthy cell and the fragmented mitochondria that result from the fission events that occur during apoptosis. Cells expressing P168A mCherry-BAX at baseline had an average mitochondrial volume of 3.3 ± 0.3 µm^3^, which was not statistically different from wild type mCherry-BAX at baseline (p = 0.23). After STS addition, the average mitochondrial volume for cells expressing P168A mCherry-BAX was 3.1 ± 0.1 µm^3^, which was statistically similar to baseline (p = 0.44). The average mitochondrial volume for cells expressing mCherry alone before and after STS induction was 1.6 ± 0.1 µm^3^ and 1.9 ± 0.1 µm^3^, respectively, which was statistically different (p < 0.05), although not significantly different from cells expressing wild type mCherry-BAX after STS addition (p = 0.36). This modest volume increase is likely not due to mitochondrial elongation induced by mCherry, but may be attributed to condensation of organelles as the cell atrophies and becomes more globular after addition of STS. Since we did not correct the 3-dimensional data for increased point spread function in the Z-plane (by deconvolution), the compression of cells may slightly change the volume measurements by changing the point spread function. To corroborate this, we also analyzed a second metric (axial length of the mitochondria) and found that there was no significant difference between cells expressing mCherry alone before and after STS addition (p = 0.14, Fig. S4). The axial length measurements also showed the same STS-induced changes in mitochondria in cells expressing either wild type mCherry-BAX (a decrease in axial length) or P168A mCherry-BAX (no change in length).

We then further examined if impaired mitochondrial fission was correlated with the cell’s ability to release cytochrome c after STS addition. Double knock-out HCT116 cells were again nucleofected with plasmids for mCherry alone, wild type mCherry-BAX, or the P168A mCherry-BAX mutant, along with plasmids expressing mito-BFP and cytochrome c-GFP. After 24 hrs, cells exhibited diffuse BAX localization and punctate cytochrome c-GFP, which co-localized with mito-BFP (Fig. S5). Cells were then treated with STS and observed 18 hrs later and quantified for diffuse localization of cytochrome c-GFP, indicating its release from the mitochondria (Figs 2O, S5). Cells expressing mCherry alone exhibited no increase in cytochrome c release at 18 hrs relative to untreated cells, while 68 ± 6% of labeled cells expressing wild type mCherry-BAX had released cytochrome c by this point (p < 0.001 relative to 0 hrs). Cells expressing the P168A mCherry-BAX mutant exhibited a modest increase in the release of cytochrome c-GFP (23 ± 8%), but this was not a significant increase over pre-addition of STS.

These data show that P168A mCherry-BAX successfully restores mitochondrial volume to similar levels as wild type mCherry-BAX, creating a condition to test the dependency of BAX on apoptosis-induced mitochondrial fission. When inducing this condition for apoptosis, both MOMP and mitochondrial fragmentation were prevented as evidenced by a reduction in cytochrome c release, and the lack of mitochondrial fragmentation. Combined with the temporal data shown in Fig. 1, this suggests a requirement for completion of the late phase of BAX recruitment, rather than as a consequence of MOMP in the early phase of BAX recruitment. Since the P168A mutant is known to impair MOMP, we cannot exclude the possibility that BAX-mediated MOMP or its associated events are required to facilitate the fragmentation process. However, BAX-dependent mitochondrial fragmentation has been reported to occur in the absence of MOMP by overexpression of both BAX and its natural antagonist, BCL-X^30^ (although BCL-X overexpression has been reported as blocking fragmentation by others^31^). Additionally, it is unlikely that caspase activation is involved in late phase fragmentation, since mitochondrial fragmentation can still occur in the presence of pan-caspase inhibitors^30^. Further experimentation is required to help distinguish if early MOMP-associated events are mechanistically relevant to the fragmentation process

It is relevant to note that there are conflicting studies of how mutations at P168 affect normal BAX function. Early studies using the P168A mutant showed that it remained cytosolic and was not translocated to the MOM unless in the presence of wild type protein^42,46^. Our previous studies of BAX recruitment kinetics supported this observation, showing that the P168A mutant was recruited to the MOM, but only after wild type protein had reached the plateau phase of its own recruitment^28^. Studies using a P168V mutant suggested that this proline was critical to allow the membrane associating α5α6 helices of BAX to become exposed^47,48^, but experiments in molecular modeling suggested that the mutant with an alanine substitution could allow for these helices to interact with the MOM^47^, opening the possibility that the P168A mutant does retain some level of normal BAX activity. Consistent with this, studies involving the expression of BAX P168A in yeast cells, or interaction of synthetic P168A BAX with lipid vesicles or isolated mitochondria (albeit in the presence of BAK) suggest that this protein can interact with lipid bilayers, form oligomers, and stimulate the release of cytochrome c^48,49^. This could explain why we see a trend toward greater cytochrome c release in double-knockout HCT116 cells after STS exposure (Fig. 2O).

### Absence or impairment of DRP1 reduces the total level of BAX recruited to mitochondria, but does not affect the timing of initiation or rate of BAX recruitment

**Figure 3:**
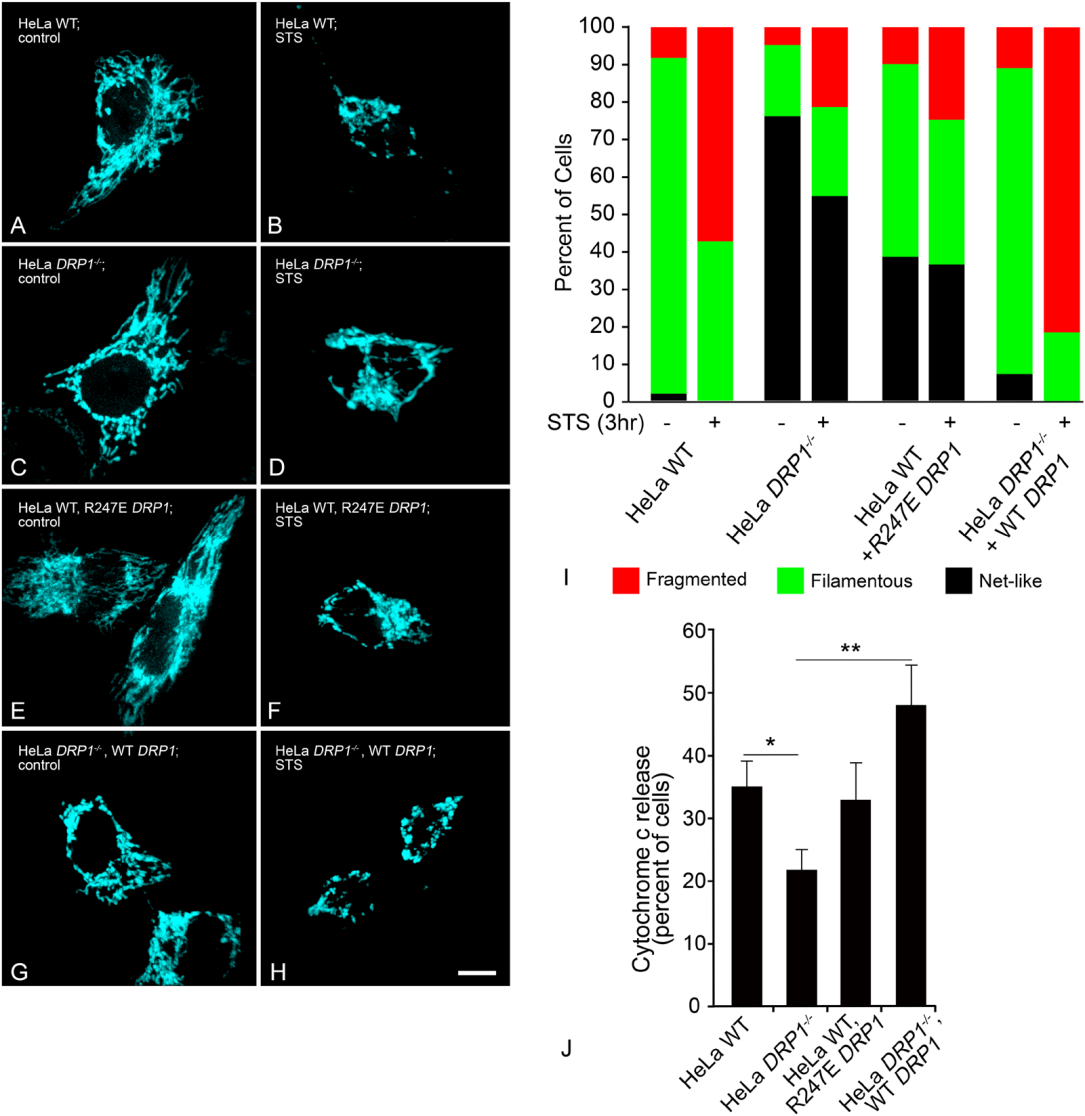
Deletion or expression of dominant negative DRP1 affects mitochondrial morphology. *DRP1*^−/−^ cells, or expression of dominant negative mutant R247E DRP1 in wild type cells, affect the ability of mitochondria to execute fission. All cells were nucleofected with a plasmid carrying mito-BFP to visualize mitochondria. Mitochondrial morphologies are shown for (**A**,**B**) HeLa wild type (WT), (**C**,**D**) HeLa^*DRP1*−/−^, (**E**,**F**) WT HeLa + R247E DRP1, or (**G**,**H**) HeLa^*DRP1*−/−^ + WT DRP1at (**A**,**C**,**E**,**G**) conditions prior to apoptosis induction, and (**B**,**D**,**F**,**H**) 3 h after addition of 1 µM staurosporine (STS), respectively. Induction of apoptosis execution was determined by presence of BAX puncta within the cell (not shown). Size bar = 5 µm. (**I**) Mitochondrial morphology was scored by a masked observer for a fragmented, filamentous, or a highly interconnected network-like appearance, before or after the addition of STS. WT cells exhibit predominantly filamentous mitochondria before STS addition, and highly fragmented mitochondria after (p < 0.0001, Chi Square test, n = 126 and 114 cells, respectively). *DRP1*^−/−^ HeLa cells exhibit significantly more net-like mitochondria (p < 0.0001 relative to WT) prior to STS addition. STS induces a modest, but significant change in mitochondrial structure toward a fragmented appearance (p = 0.001, n = 144 and 123 cells, respectively). The expression of the dominant-negative R247E DRP1 mutant induces an intermediate phenotype for mitochondria, with a predominant mixture of filamentous and net-like structures that is significantly different from both WT cells (p < 0.0001) and *DRP1*^−/−^ cells (p < 0.0001) prior to STS addition. The addition of STS promotes a shift toward fragmented mitochondria (p = 0.015, n = 81 and 81 cells, respectively). Expression of WT DRP1 in *DRP1*^−/−^ cells reverts pre-STS mitochondrial morphology to a phenotype that is statistically similar to WT HeLa cells (p = 0.163), and the addition of STS is accompanied by a significant increase in mitochondrial fragmentation (p < 0.0001, n = 99 and 90 cells, respectively), that is even more pronounced than in WT cells (p < 0.001). (**J**) In a different set of experiments, we also examined the effect of DRP1 deficiency on the release of cytochrome c. Cells of the four different groups were nucleofected with cytochrome c-GFP, treated with STS, and then scored for cytosolic or mitochondrial cytochrome c-GFP. The graph shows the percentage of scored cells (mean ± s.e.m.) that exhibit the release of cytochrome c, 3 h after STS addition. Consistent with other reports, *DRP1*^−/−^ cells exhibit retarded release of cytochrome c relative to both WT cells (*p < 0.01, *t*-test) or *DRP1*^−/−^ cells expressing a WT DRP1 construct (**p < 0.0002). WT cells expressing the R247E mutant have an intermediate phenotype that is statistically similar to both WT and *DRP1*^−/−^ cells (p = 0.693 and 0.095, respectively), similar to the mitochondrial morphology phenotype. Total cells scored, n = 232 (WT), 215 (*DRP1*^−/−^), 136 (WT cells + R247E DRP1), and 122 (*DRP1*^−/−^ cells + WT DRP1).

**Figure 4:**
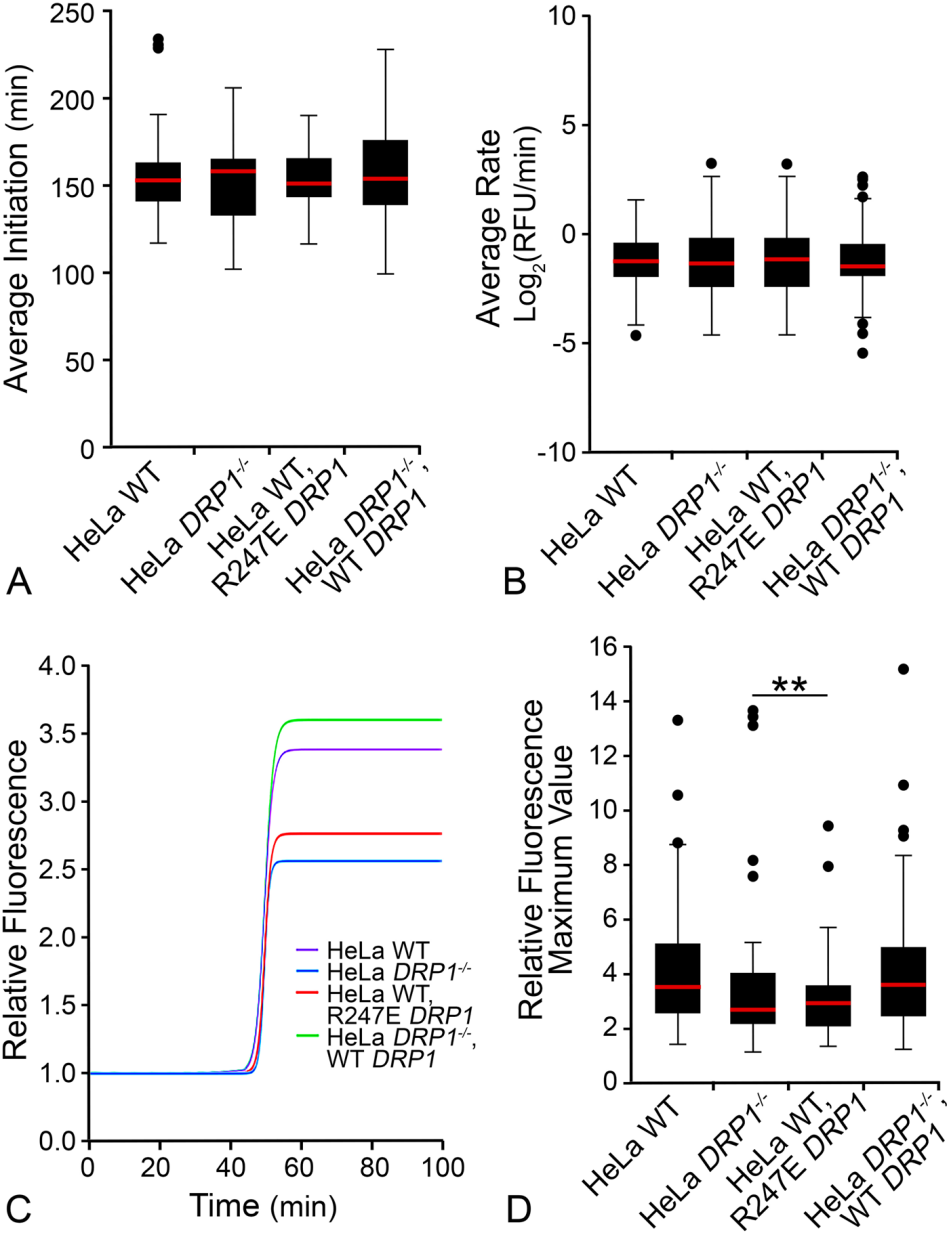
Deletion or expression of dominant negative DRP1 affects total BAX recruitment. Box and whisker plots show the (**A**) BAX recruitment initiation time (from staurosporine addition) and (**B**) rate of BAX recruitment. There were no significant differences among all conditions for either the initiation or rate of BAX recruitment (p = 0.75, p = 1.0, respectively, by one-way ANOVA.) (**C**) BAX recruitment sigmoid curves defined by median optimal parameters for each set of BAX recruitment curves was plotted for each condition (see Fig. S7 for complete set of curves). The median plateau value (Y-max) for BAX recruitment is reduced by 25% in cells lacking DRP1 and 20% in cells expressing the R247E DRP1 dominant-negative mutant, respectively. (**D**) Box and whisker plot of Y-max values reveal that HeLa wild type cells and HeLa^*DRP1*−/−^ cells expressing exogenous WT DRP1 have significantly less BAX recruited than HeLa^*DRP1*−/−^ cells and HeLa cells expressing R247E DRP1 (**p < 0.01).

DRP1 is another important player in mitochondrial dynamics of healthy cells. An imbalance of the mitochondrial equilibrium through DRP1 knockdown, or expression of dominant-negative mutants, creates a contiguous network of large, multi-fasciculated organelles^14,16,34,50,51^. During apoptosis, loss of DRP1 prevents mitochondrial fragmentation^16,50,52^, and has been reported to delay cytochrome c release and BAX oligomerization^16,17,34,53^. However, these conclusions were reached using only static measurements. Given the lack of temporal data describing the influence of DRP1 on BAX recruitment and mitochondrial remodeling, we next evaluated the kinetics of BAX recruitment as a function of wild type, ablated or impaired DRP1 activity. For these experiments, we used HeLa cells, which either had been engineered to be deficient for DRP1 (HeLa^*DRP1*−/−^) or were expressing the R247E dominant negative mutant for DRP1^34^. Both conditions showed significantly greater percentages of cells exhibiting a hyperfused mitochondrial network when compared to wild type HeLa cells or HeLa^*DRP1*−/−^ cells that had been rescued by nucleofection with a plasmid containing wild type DRP1 (Fig. 3A–H). We then evaluated if the hyperfused network of mitochondria in HeLa^*DRP1*−/−^ cells or wild type HeLa cells expressing R247E DRP1 was maintained after 3 hours STS treatment. While STS did induce a significant redistribution of mitochondrial morphology in these cells, including an increase in the number of cells with fragmented mitochondria (relative to pre-STS treatment, p = 0.001 and 0.015, respectively), both wild type and rescued HeLa cells exhibited a substantially greater increase in fragmentation under these conditions (Fig. 3I, p < 0.0001). Assessment of cytochrome c release showed that among four DRP1 conditions, only HeLa^*DRP1*−/−^ cells showed a significant reduction in the percentage of cells with cytochrome c release at this timepoint (Fig. 3J, p = 0.01 and 0.0001, relative to wild type and rescued cells, respectively). HeLa cells expressing the R247E dominant negative mutant represented what could be considered an intermediate phenotype, being not statistically different from either wild type, rescued, or *DRP1*^−/−^ cells. Collectively, these data are consistent with the observations of other groups showing both delayed MOMP and fission in DRP1-impaired cells^17,34,50,53^. When we examined the kinetics of BAX recruitment in cells with impaired DRP1 function, however, we observed that neither the average time of initiation, nor the average rate of BAX recruitment was affected in either HeLa^*DRP1*−/−^ cells, or wild type HeLa cells expressing the R247E DRP1 mutant, when compared to wild type cells or rescued HeLa^*DRP1*−/−^ cells (Fig. 4A,B). DRP1-deficiency also had no apparent effect on the activation of recruited endogenous BAX, which could be stained positive using the 6A7 monoclonal antibody (Fig. S6A–D). We did observe, however, a significant reduction in the total level of BAX recruited to the MOM in kinetic analysis of cells with absent or impaired DRP1. Figure 4C shows the median recruitment curves of all the mitochondrial foci examined in each condition (see Fig. S7 for complete datasets), and Fig. 4D shows a graphical representation of the combined data on final recruitment levels. HeLa^*DRP1*−/−^ cells exhibited 25% less BAX recruitment and R247E DRP1 expressing cells showed 20% less recruited BAX (p < 0.01 and p < 0.001, respectively compared to wild type HeLa cells). The decrease in total BAX recruitment could be completely rescued in HeLa^*DRP1*−/−^ cells expressing an exogenous wild type DRP1 construct (p = 0.84, compared to wild type cells). This data was corroborated in static experiments assessing the level of staining intensity for the endogenous BAX 6A7 antigen in *DRP1*^−/−^ HeLa cells, which showed a 25.6% reduction in activated BAX per punctum on average and a reduction in the average number of BAX foci formed, relative to wild type cells (Fig. S6E,F). This was not caused by a reduction in the overall amount of BAX being expressed in HeLa^*DRP1*−/−^ cells (Fig. S6G,H).

These results align with reports that the membrane tethering activity of DRP1 acts to promote BAX oligomerization^34^, but show that DRP1 is not required for the initial MOM recruitment or activation of BAX during apoptosis. We speculate that initiation of the MOMP activity of BAX is independent of the function of DRP1, but that DRP1 facilitates further accumulation of BAX in a later phase of recruitment. Consistent with this concept, DRP1 is not required for the release of SMAC/Diablo, another mitochondrial protein that requires BAX permeabilization of the mitochondrial membrane^52,54,55^, while the impaired release of cytochrome c may be more related to the effect of DRP1 on cardiolipin transfer from the mitochondrial inner membrane to the MOM^56^. Further accumulation of BAX, mediated by DRP1, may contribute to so-called “non-random BAX structures”^57^, which have been described as BAX-containing lines, arcs, and rings in studies using super resolution microscopy. The formation of these non-random structures is reduced in DRP1-deficient cells^54^, which correlates with a delay in cytochrome c release. Conversely, there is clearly an intrinsic ability of BAX to form “arcs and rings”, since similar ring structures are formed by purified BAX (in the presence of tBID) in cardiolipin-containing liposomes^58^. Probably more relevant, however, is that non-random BAX structures underlie the formation of macropores necessary for mitochondrial inner membrane herniations and the release of mtDNA^38^.

**Figure 5:**
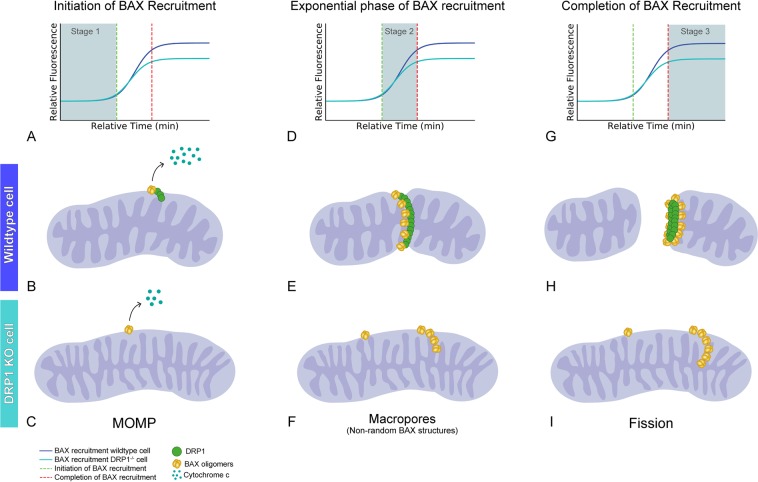
Proposed model for BAX participation in mitochondrial fission during apoptosis. (**A**–**C**) Initiation of BAX recruitment (Stage 1) is comprised of BAX integration into the MOM, MOMP and cytochrome c release. At this stage, (**A**) the time to initiate BAX recruitment is indistinguishable between wild type and *DRP1*^−/−^ cells. In wild type cells, DRP1 has already recruited to the MOM. (**B**,**C**) Cytochrome c is released in both cell types, however *DRP1*^−/−^ cells show a reduction in the percentage of cells releasing cytochrome c. This difference may be related to a proposed function of DRP1 in facilitating transfer of cardiolipin, which is associated with cytochrome c, from the inner membrane to the MOM^51^. The release of other molecules, such as SMAC, is not inhibited in *DRP1*^−/−^ cells^48–50^. BAX dimers and/or small oligomers are present at this stage. We define BAX structures at this stage as micropores. (**D**–**F**) The exponential phase of BAX recruitment (Stage 2) is defined as the period of BAX accumulation at the MOM. (**D**) The rate of BAX recruitment does not differ between wild type and *DRP1*^−/−^ cells. (**E**) At the site of DRP1 constriction of the mitochondria in wild type cells, BAX accumulation takes on higher order non-random structures, however (**F**) these structures lack organized localization at the scission site in the absence of DRP1. We define these BAX structures as macropore oligomers, and may be required for the release of larger molecules and/or evulsions of the mitochondrial inner membrane. (**G**–**I**) Completion of BAX recruitment (Stage 3) is identified as the point where the (**G**) BAX recruitment curve plateaus. (**H**) Importantly, this turning point is accompanied by mitochondrial fragmentation in wild type cells. DRP1-mediated recruitment of BAX to the mitochondrial scission sites allows for non-random BAX structures or rings that are organized to completely encircle the mitochondrion. This likely weakens membrane integrity through presence of many adjacent macropores, creating a perforated membrane, and allowing constriction pressure created by DRP1 and its assemblies to complete the membrane separation. We define these BAX structures as fission oligomers. This model, where BAX perforates the membrane, is consistent with a model originally proposed by Martinou and Youle^29^. (**I**) Conversely, BAX structures on DRP1-deficient mitochondria are unable to organize around DRP1 identified scission sites, therefore cannot provide a perforated region to facilitate mitochondrial fragmentation. Loss or impairment of DRP1 function also results in fewer BAX puncta that contain approximately 25% less BAX protein. Therefore, DRP1-mediated fission oligomers may evolve both by coalescence of macropore oligomers and late-stage recruitment of inactive latent BAX monomers. For example, P168A mutant BAX cannot normally recruit to the MOM in apoptotic cells, unless in the presence of wild type BAX protein^42^. Kinetic studies indicate that this recruitment process occurs only after wild type BAX nears the plateau phase of its recruitment^28^, when we predict that fission oligomers are being formed.

While a great deal of speculation has been posited regarding the formation of non-random BAX structures and their role as pores in the MOM, the focus of this study was to evaluate BAX function as a vital component in the process of mitochondrial fission. Careful quantification of BAX containing structures by Salvador-Gallego and colleagues revealed that non-random structures represented only a small percentage of the total BAX that had accumulated at the MOM^57^. A majority of BAX formed small “dots” or large aggregates. Aggregates were observed to often form at discrete locations on mitochondrial tubules, or were identified at the tips of mitochondrial fragments^54,57^, possibly at a site of a recent fission event, such as described by Karbowski and colleagues^31^, and demonstrated in Fig. 1. Similarly, non-random BAX structures may also participate in fission events. Große and colleagues^54^ rarely observed rings, and then only small ones, on elongated mitochondria, but often found rings associated with mitochondrial fragments. BAX-containing lines were also described as “wrapping” around mitochondria by Salvador-Gallego and colleagues^57^. Given such observations, it is not difficult to envision a model where larger BAX assemblies could help perforate a mitochondrion to weaken membrane integrity, allowing other members of the fission machinery to more efficiently complete the process (Fig. 5). We speculate that the construction of these assemblies is mediated by DRP1. This concept is consistent with results obtained in protozoan studies using *T. brucei*. Fission of the single giant mitochondrion in this organism is stimulated when an exogenous BAX protein is introduced, but only in the presence of the endogenous dynamin-like protein^36,59^.

In addition to DRP1, DYN2 is also necessary to execute the process of mitochondrial fission^17^. *In vitro* studies have shown that while DRP1 can tubulate membranes, it has no intrinsic ability to complete scission of them^60^. Alternatively, this ability is intrinsic to classical dynamin proteins^61^. Kinetic studies show accumulation of DRP1 prior to DYN2 at mitochondrial fission sites, suggesting that DRP1 may facilitate recruitment of DYN2 to these regions, similar to the mechanism we are proposing for BAX aggregation. Loss of DYN2 expression exerts similar effects as loss of DRP1, such as elongated mitochondria in living cells and a delayed onset of apoptosis. Knockdown of DRP1 or DYN2 with siRNA reduced the level of cytochrome c release when compared to control after 1.5 hours of STS treatment, but the same conditions showed accelerated BAX activation compared to control^17^ when assessed by exposure of the 6A7 antibody epitope. These data, which show that BAX recruitment and activation are not strictly reliant on DRP1, are consistent with our findings that BAX recruitment initiates at the same time after STS addition in wild type and DRP1-deficient cells HeLa cells (Fig. 4B).

In summary, we directly tested the requirement of BAX in the apoptosis-associated fission process by using the P168A BAX mutant, to first recover mitochondrial morphology, then to show lack of mitochondrial fragmentation after apoptosis induction. In kinetic studies we show that the completion of BAX recruitment identifies the timeframe for the start of rapid mitochondrial fragmentation, a phase of BAX recruitment that previously had no defined role. Additionally, this phase of BAX recruitment is affected by the loss or mutation of DRP1, suggesting a cooperative role in the rapid mitochondrial fragmentation process during apoptosis, consistent with a model originally proposed by Martinou and Youle^29^ where BAX may perforate the MOM through the formation of hemifusion intermediates to facilitate the fragmentation. Our prediction is that BAX oligomerization has an early phase, creating micropores leading to MOMP, an intermediate phase creating macropores leading to the release of larger mitochondrial components (such as evulsions of the inner membrane), and a late phase facilitated by DRP1, that is associated with mitochondrial fragmentation. Ultimately, identifying BAX mutants that can distinguish between these phases is a necessary step in confirming this model.

## Materials and Methods

### Tissue culture cells and nucleofection

All tissue culture cells were maintained at 37 °C and 5% CO_2_. D407 cells (immortalized human retinal pigment epithelial cells), 661W cells (differentiated mouse neuron progenitor cells) and HeLa cells (immortalized human cervical cancer cells) were cultured in DMEM (Cellgro, Mediatech, Inc., Manassas, VA) supplemented with 1% Antibiotic/Antimycotic (Cellgro, Mediatech, Inc., Manassas, VA) and 3% (D407) or 10% (661W, HeLa) Fetal Bovine Serum (Atlanta Biologicals, Norcross, GA). 661W cells were differentiated into a neuron-like phenotype by treatment with 316 nM staurosporine for 24 hours. HCT116 cells (immortalized human colorectal cancer cells) were cultured in McCoy’s 5A medium (Lonza, Basel, Switzerland) supplemented with 10% FBS (Cellgro) and 1% Antibiotic/Antimycotic (Cellgro). ARPE-19 cells (immortalized human retinal pigment epithelial cells) were cultured in DMEM:F12 (Hyclone, GE Healthcare Life Sciences, Marlborough, MA), supplemented with 10% Fetal Bovine Serum (Atlanta Biologicals) and 1% Antibiotic/Antimycotic (Cellgro). 1 μg of each plasmid DNA was used for each nucleofection reaction (Amaxa Nucleofector, Lonza, Basel, Switzerland), which was comprised of one million cells unless otherwise noted. D407, ARPE-19, HeLa, and HCT116 cells were induced for apoptosis using a final concentration of 1 µM staurosporine (Sigma, St. Louis, MO) prepared in DMSO, while overexpression of Flag-HDAC3 was used to induce apoptosis in 661W cells (Addgene, Cambridge, MA, plasmid #13819), a gift from Eric Verdin. HDAC3 is selectively toxic to differentiated neurons^62^.

D407 and ARPE-19 cells were a gift from Dr. Aparna Lakkaraju. The D407 cells originated from Dr. R. Hunt at the University of South Carolina. HCT116^*BAX*−/−*/BAK*−/−^ cells and HeLa^*DRP1*−/−^ cells were a gift from Dr. Richard Youle^41^. Experiments introducing BAX mutant DNA, which prevented cytochrome c release after STS treatment, provided evidence of authenticity for HCT116^*BAX*−/−*/BAK*−/−^ cells. HeLa^*DRP1*−/−^ cells stocks were authenticated in Dr. Youle’s laboratory prior to receipt. 661W cells were a gift from Dr. Leonard Levin and have been confirmed in our laboratory as *Mus musculus* origin by PCR of the mitochondria d-loop^63^, and their ability to differentiate into a neuron-like phenotype after exposure to 316 nM STS by extending neurite like processes and the up-regulation of neuronal markers^64^.

### Immunostaining for activated BAX

Three hours after the addition of 1μM staurosporine, wild type and *DRP1*^−/−^ HeLa cells were fixed in 4% paraformaldehyde in phosphate buffered saline (PBS) for 8 minutes at room temperature. Cells were then washed three times in PBS for five minutes before blocking in 10% horse serum and 0.3% Triton X-100 in PBS for 1 hour at room temperature. After washing three times in PBS for five minutes, cells were incubated in 5% horse serum with 0.1% Triton X-100 and 1:200 anti-BAX (6A7) mouse monoclonal antibody (catalog #sc-23959, Santa Cruz Biotechnology, Santa Cruz, CA) for 24 hours at 4 °C. Cells were again washed and incubated in 5% horse serum and 0.1% Triton X-100 containing 1:1000 Alexa Fluor 488-congugated AffiniPure goat anti-mouse antibody (catalog #115-545-003, Jackson ImmunoResearch, West Grove, PA) for 2 hours at room temperature. Slides were then washed with PBS and mounted with Immunomount (ThermoFisher Scientific, Waltham, MA).

Slides were imaged using a Zeiss Axioplan 2 Imaging microscope with Axiovision 4.6.3.0 software (Carl Zeiss MicroImaging Inc., Dublin, CA). All images were obtained using a 63X oil objective with the same laser intensity (50%) and exposure time (500 ms). Fluorescence intensity was measured using ZEN 2.3 (blue edition) imaging software (Carl Zeiss MicroImaging, Inc.). Twenty puncta from each cell (n = 5 per group) were outlined as well as twenty spots from the background of the image. The average intensity of the background of each image was subtracted from the intensity of each puncta.

### Western blotting

HeLa wild type and *DRP1*^−/−^ cells were washed with PBS, pelleted, and frozen at −80 °C. Cell pellets were resuspended in 200 µl of RIPA buffer (50 mM Tris, pH 8.0, 150 mM NaCl, 5 mM EDTA, 1.0% IGEPAL, 0.5% Na Deoxycholate, 0.1% SDS) containing 0.1% PMSF and sonicated. Protein levels were quantified using the Pierce bicinchoninin acid assay (BCA, ThermoFisher Scientific) and 20, 30, and 50 µg of protein from each cell pellet was combined with 2x Laemmli buffer (125 mM Tris, pH 6.8, 4% SDS, 10% glycerol, 10% β-mercaptoethanol, 0.004% bromphenol blue), heated for 5 min at 70 °C, and run on 12% polyacrylamide SDS gels. After electrophoresis, proteins were electroblotted onto Immobilon-FL PVDF transfer membrane (EMD Millipore, ThermoFisher Scientific). Total protein loads were measured by staining the membranes with Revert 700 total protein stain (LI-COR, Lincoln, NB) and imaged using a LI-COR Odyssey CLX scanner at 700 nm. Transfer membranes were then blocked in PBS containing 0.1% Tween-20 (PBST) and 5% powdered milk for 2 hours at 22 °C. BAX was detected by incubating the membrane in PBST containing a rabbit polyclonal antibody against BAX (#B3428, 1:500 dilution, Sigma) overnight at 4 °C. This antibody was previously confirmed to be BAX-specific by the absence of a signal in tissue extracts from *Bax*-deficient mice compared to wild type tissues^65^. After washing in PBST, the blots were then incubated in PBST with 5% milk containing goat anti-rabbit IgG conjugated to IRDye800CW (#926-32211, 1:5000 dilution, LI-COR) for 2 hours at 26 °C. Membranes were then washed in PBST and imaged on the Odyssey CLX scanner at 800 nm. For quantification, LI-COR scans were converted to greyscale and pixel density was measured using ImageJ (v1.42q). To normalized BAX levels, the pixel level of BAX was divided by the pixel value for total protein in each lane, for each different concentration of protein loaded. Relative values for all 3 measurements for two separate cell pellets of each genotype were graphed and used for statistical analysis.

### Plasmids

GFP-BAX, mCherry-BAX, the transmembrane mutant mCherry-BAXP168A, and mito-BFP were cloned in the lab as previously described^28,66^. The vector backbone for these plasmids include peGFP-C3 and pmCherry-C1 (Clontech, Mountainview, CA) and TagBFP-N (Evrogen, Moscow, Russia). All transgenes were expressed from the CMV immediate early promoter. Cytochrome c-GFP (Addgene, plasmid #41182) was a gift from Douglas Green^67^. mCherry-DRP1 (Addgene, plasmid #49152) was a gift from Dr. Gia Voeltz^68^ and was used to generate the DRP1 mutant (R247E mCherry-DRP1) construct using the QuikChange II site-directed mutagenesis protocol (Agilent Technologies, Santa Clara, CA) to mutate arginine 247 to glutamic acid, which was verified by sequencing.

### Image acquisition and analysis

Live-cell and static imaging was performed using the Andor Revolution XD spinning disc confocal microscopy system (Andor, Belfast, Northern Ireland) comprised of the Nikon Eclipse Ti inverted microscope, Nikon objectives, the iXon x3 897 EM-CCD camera, a Yokogawa CSU-X1 confocal spinning disk head, the Andor Laser Combiner with four solid state lasers, an ASI motorized stage with Piezo-Z, and an Okolab CO_2_ cage incubator for temperature and CO_2_ control at 37 °C and 5% CO_2_. For time-lapse imaging, cells were plated at a density of 100,000–300,000 cells per well on 4-well chamber slides containing #1.5 optical grade plastic (Ibidi, Madison, WI). Live-cell treatments were staggered for an appropriate time for the cell type to ensure the ability to image each well on a slide. Prior to imaging, phenol-containing media was removed from the well, and replaced with a house made ‘recording media’ (HBSS with 1.26 mM calcium chloride, 0.49 mM magnesium chloride, 0.4 mM magnesium sulfate, no phenol red, 4.5 g/L glucose and 10 mM Hepes) supplemented with appropriate serum concentration. Under temperature and CO_2_ control, time-lapse imaging was performed using a 100X oil objective (numerical aperture = 1.49, temperature collar for 23 °C to 37 °C) by collecting z-stacks consisting of 20–25 optical sections at 0.22 µm (Nyquist) taken every minute for one to two hours. All imaging was performed under the same laser intensity, electron-multiplying gain and exposure time. All time-lapse images were subject to the same background subtraction and Gaussian filter within the IMARIS 7.7 software (Bitplane, Concord, MA), where each filter has its own constant algorithm and threshold setting. ARPE-19 cells were analyzed with IMARIS 9.2.1 under the same conditions.

From each cell captured using time-lapse live-imaging, BAX recruitment was analyzed as described below. A more detailed description is available in a previous publication^28^. First, isolated mitochondria organelles were identified based on mito-BFP label using the ‘spots’ function in IMARIS. From each spot, the fluorescence intensity of tagged-BAX was collected over the duration of the time-lapse image. Fluorescence curves from several spots were collected for each cell. All fluorescence intensity curves were normalized to their own baseline fluorescence intensity to allow for comparison among curves. Next, a sigmoid curve was fit to the normalized fluorescence data using the modified sigmoid equation below (Eq. 1). Parameters (*a*, *b*, *k*, *x*_0_, *y*_0_) were tested to find the best fit curve using a linear regression model in the SciPy library.

1 $${\rm{y}}=\frac{a}{b+{e}^{-k(x-{x}_{0})}}+{y}_{0}$$

This best fit curve was defined as a BAX recruitment curve, which contained optimal parameters ($a,{b},{k},{x}_{0},{y}_{0}$) from which several metrics for analysis could be derived. First, the rate of BAX recruitment was defined as the maximum rate of the sigmoid curve. BAX recruitment initiation was defined from the 5% threshold value, which is where the slope of a line (derived the maximum rate of the sigmoid curve) intersects the minimum boundary of the sigmoid curve. (See Fig. S1). For compiled BAX recruitment curves, the optimal parameters were collected for each sigmoid curve from the population of mitochondria for each condition. The median optimal parameters for each treatment group was used to plot a single, representative sigmoid curve.

Mitochondrial volume measurements were obtained from the “surfaces” function in IMARIS. In time-lapse volume measurements, the mito-BFP channel was normalized using the ‘Normalize time points’ Xtension in IMARIS to correct for photobleaching throughout the duration of the time-lapse video. For static volume measurements, the IMARIS-defined “surfaces” encompassing mitochondria were manually edited on a per cell basis to acquire precise volume measurements, rather than using a threshold approach as in time-lapse image analysis, which allows for comparison among timepoints. All images within a single figure were prepared using the same brightness, contrast and color adjustments in Photoshop. To allow for best visualization, cells of interest were cropped from original image files containing multiple cells and placed on a black background.

### Electron Microscopy

HCT116 cells were grown on round coverslips in a 12-well tissue culture dish. Cells were prepared using a protocol optimized to preserve mitochondrial structure similar to the protocol used by Kim and colleagues^69^. Briefly, cells were fixed in 2% paraformaldehyde and 2.5% glutaraldehyde in 0.1 M cacodylate buffer for 5 min at room temperature, then continued fixation for 30 minutes on ice. The wash buffer, post-fix, water and uranyl acetate for the following steps were kept ice-cold to allow for optimal mitochondrial preservation. A wash buffer containing 0.1 M sodium cacodylate and 3 mM calcium chloride was used for a series of 5 washes. Washes were followed by a 30 minute post-fixation on ice. The post-fix was comprised of 1% osmium tetroxide, 0.8% potassium ferrocyanide, and 3 mM calcium chloride. The cells were then washed with distilled water and stained with 2% uranyl acetate overnight. The following day, cells were dehydrated, infiltrated and embedded in Epon epoxy and sectioned at 60–90 nm. Sections were imaged on a Phillips CM120 transmission electron microscope (FEI Co., Hillsboro, OR). Measurements of mitochondrial area were taken from round sections, since we interpreted these as being truer to cross-sectional area and reduced bias from longitudinal sections. Area was measured using ImageJ (v1.42q).

### Mitochondrial network categorization

Net-like mitochondria were classified as those exhibiting near complete connectivity of the mitochondrial network, often with bulbous protrusions at intersections of several filamentous regions. Mitochondria networks classified as filamentous were identified by their more elongated structure, but with clear separation among groupings of filamentous structures. Sometimes these cells contained smaller organelles. Fragmented mitochondria networks were identified by their more dotted or rice-shaped organelle appearance, with few if any elongated spaghetti-like structures.

### Statistical analysis

Time-lapse mitochondria volume measurements were compared based on the average normalized volume for 20 minutes before BAX initiation, during the recruitment phase, and 20 minutes after BAX recruitment completion. The average normalized volumes from each phase were compared using two-tailed student t-test. For mitochondria volume comparisons, fragmentation, and cytochrome c release comparisons, percentages of total cells from three independent experiments were tested using two-tailed student t-test. For calculated statistics based per cell, the standard deviation was calculated for an individual cell and expressed as a percentage of the mean. For analysis of mitochondrial morphology in DRP1-deficient cells, the different proportions of fragmented, filamentous, and net-like mitochondria were compared between groups using a Chi square test. Significant differences were set at a p value < 0.05. Comparisons of BAX recruitment rates and initiation times among the four conditions in Fig. 4 were calculated using a one-way ANOVA.

## Supplementary information

Supplemental Video 1

Supplemental Info

## Data Availability

The datasets generated during and analyzed during the current study are available from the corresponding author on reasonable request. Detailed methods for BAX recruitment analysis can be referenced in a previous publication^28^ and corresponding python scripts are available in the public repository (https://github.com/maggiemaes/Methods-for-BAX-recruitment-analysis).

## References

[1] Oltvai ZN, Milliman CL, Korsmeyer SJ (1993). Bcl-2 heterodimerizes *in vivo* with a conserved homolog, Bax, that accelerates programmed cell death. Cell.

[2] Czabotar PE (2013). Bax crystal structures reveal how BH3 domains activate Bax and nucleate its oligomerization to induce apoptosis. Cell.

[3] Hsu YT, Youle RJ (1997). Nonionic detergents induce dimerization among members of the Bcl-2 family. J Biol Chem.

[4] Suzuki M, Youle RJ, Tjandra N (2000). Structure of Bax: coregulation of dimer formation and intracellular localization. Cell.

[5] Hsu YT, Wolter KG, Youle RJ (1997). Cytosol-to-membrane redistribution of Bax and Bcl-X(L) during apoptosis. Proc Natl Acad Sci USA.

[6] Wolter KG (1997). Movement of Bax from the cytosol to mitochondria during apoptosis. J Cell Biol.

[7] Antonsson B, Montessuit S, Sanchez B, Martinou JC (2001). Bax is present as a high molecular weight oligomer/complex in the mitochondrial membrane of apoptotic cells. J Biol Chem.

[8] Zhang Z (2016). BH3-in-groove dimerization initiates and helix 9 dimerization expands Bax pore assembly in membranes. Embo J.

[9] Saito M, Korsmeyer SJ, Schlesinger PH (2000). BAX-dependent transport of cytochrome c reconstituted in pure liposomes. Nat Cell Biol.

[10] Antonsson B, Montessuit S, Lauper S, Eskes R, Martinou JC (2000). Bax oligomerization is required for channel-forming activity in liposomes and to trigger cytochrome c release from mitochondria. Biochem J.

[11] Chang LK, Putcha GV, Deshmukh M, Johnson EM (2002). Mitochondrial involvement in the point of no return in neuronal apoptosis. Biochimie.

[12] Karbowski M, Youle RJ (2003). Dynamics of mitochondrial morphology in healthy cells and during apoptosis. Cell Death Differ.

[13] Youle RJ, Karbowski M (2005). Mitochondrial fission in apoptosis. Nat Rev Mol Cell Biol.

[14] Karbowski M (2004). Quantitation of mitochondrial dynamics by photolabeling of individual organelles shows that mitochondrial fusion is blocked during the Bax activation phase of apoptosis. J Cell Biol.

[15] Hoppins S, Lackner L, Nunnari J (2007). The machines that divide and fuse mitochondria. Annu Rev Biochem.

[16] Frank S (2001). The role of dynamin-related protein 1, a mediator of mitochondrial fission, in apoptosis. Dev Cell.

[17] Lee JE, Westrate LM, Wu H, Page C, Voeltz GK (2016). Multiple dynamin family members collaborate to drive mitochondrial division. Nature.

[18] Suzuki M, Jeong SY, Karbowski M, Youle RJ, Tjandra N (2003). The solution structure of human mitochondria fission protein Fis1 reveals a novel TPR-like helix bundle. J Mol Biol.

[19] Palmer CS (2011). MiD49 and MiD51, new components of the mitochondrial fission machinery. EMBO Rep.

[20] Hinshaw JE (2000). Dynamin and its role in membrane fission. Annu Rev Cell Dev Biol.

[21] Olichon A (2003). Loss of OPA1 perturbates the mitochondrial inner membrane structure and integrity, leading to cytochrome c release and apoptosis. J Biol Chem.

[22] Chen H (2003). Mitofusins Mfn1 and Mfn2 coordinately regulate mitochondrial fusion and are essential for embryonic development. J Cell Biol.

[23] Hoppins S (2011). The soluble form of Bax regulates mitochondrial fusion via MFN2 homotypic complexes. Mol Cell.

[24] Cleland MM (2011). Bcl-2 family interaction with the mitochondrial morphogenesis machinery. Cell Death Differ.

[25] Karbowski M, Norris KL, Cleland MM, Jeong SY, Youle RJ (2006). Role of Bax and Bak in mitochondrial morphogenesis. Nature.

[26] Kraus F, Ryan MT (2017). The constriction and scission machineries involved in mitochondrial fission. J Cell Sci.

[27] Capano M, Crompton M (2002). Biphasic translocation of Bax to mitochondria. Biochem J.

[28] Maes ME, Schlamp CL, Nickells RW (2017). Live-cell imaging to measure BAX recruitment kinetics to mitochondria during apoptosis. PLoS One.

[29] Martinou JC, Youle RJ (2011). Mitochondria in apoptosis: Bcl-2 family members and mitochondrial dynamics. Dev Cell.

[30] Sheridan C, Delivani P, Cullen SP, Martin SJ (2008). Bax- or Bak-induced mitochondrial fission can be uncoupled from cytochrome C release. Mol Cell.

[31] Karbowski M (2002). Spatial and temporal association of Bax with mitochondrial fission sites, Drp1, and Mfn2 during apoptosis. J Cell Biol.

[32] Basanez G (2002). Bax-type apoptotic proteins porate pure lipid bilayers through a mechanism sensitive to intrinsic monolayer curvature. J Biol Chem.

[33] Garcia-Saez AJ (2005). Peptides derived from apoptotic Bax and Bid reproduce the poration activity of the parent full-length proteins. Biophys J.

[34] Montessuit S (2010). Membrane remodeling induced by the dynamin-related protein Drp1 stimulates Bax oligomerization. Cell.

[35] Arnoult D, Grodet A, Lee YJ, Estaquier J, Blackstone C (2005). Release of OPA1 during apoptosis participates in the rapid and complete release of cytochrome c and subsequent mitochondrial fragmentation. J Biol Chem.

[36] Esseiva AC (2004). Temporal dissection of Bax-induced events leading to fission of the single mitochondrion in Trypanosoma brucei. EMBO Rep.

[37] Nechushtan A, Smith CL, Lamensdorf I, Yoon SH, Youle RJ (2001). Bax and Bak coalesce into novel mitochondria-associated clusters during apoptosis. J Cell Biol.

[38] McArthur Kate, Whitehead Lachlan W., Heddleston John M., Li Lucy, Padman Benjamin S., Oorschot Viola, Geoghegan Niall D., Chappaz Stephane, Davidson Sophia, San Chin Hui, Lane Rachael M., Dramicanin Marija, Saunders Tahnee L., Sugiana Canny, Lessene Romina, Osellame Laura D., Chew Teng-Leong, Dewson Grant, Lazarou Michael, Ramm Georg, Lessene Guillaume, Ryan Michael T., Rogers Kelly L., van Delft Mark F., Kile Benjamin T. (2018). BAK/BAX macropores facilitate mitochondrial herniation and mtDNA efflux during apoptosis. Science.

[39] Xu XP (2013). Three-dimensional structure of Bax-mediated pores in membrane bilayers. Cell Death Dis.

[40] Gillies LA (2015). Visual and functional demonstration of growing Bax-induced pores in mitochondrial outer membranes. Mol Biol Cell.

[41] Wang C, Youle RJ (2012). Predominant requirement of Bax for apoptosis in HCT116 cells is determined by Mcl-1’s inhibitory effect on Bak. Oncogene.

[42] Valentijn AJ, Upton JP, Bates N, Gilmore AP (2008). Bax targeting to mitochondria occurs via both tail anchor-dependent and -independent mechanisms. Cell Death Differ.

[43] Edlich F (2011). Bcl-x(L) retrotranslocates Bax from the mitochondria into the cytosol. Cell.

[44] Delivani P, Adrain C, Taylor RC, Duriez PJ, Martin SJ (2006). Role for CED-9 and Egl-1 as regulators of mitochondrial fission and fusion dynamics. Mol Cell.

[45] Brooks C (2007). Bak regulates mitochondrial morphology and pathology during apoptosis by interacting with mitofusins. Proc Natl Acad Sci USA.

[46] Schinzel A (2004). Conformational control of Bax localization and apoptotic activity by Pro168. J Cell Biol.

[47] Cartron PF (2005). Distinct domains control the addressing and the insertion of Bax into mitochondria. J Biol Chem.

[48] Simonyan L (2017). The substitution of Proline 168 favors Bax oligomerization and stimulates its interaction with LUVs and mitochondria. Biochim Biophys Acta Biomembr.

[49] Arokium H, Camougrand N, Vallette FM, Manon S (2004). Studies of the interaction of substituted mutants of BAX with yeast mitochondria reveal that the C-terminal hydrophobic alpha-helix is a second ART sequence and plays a role in the interaction with anti-apoptotic BCL-xL. J Biol Chem.

[50] Otera H, Miyata N, Kuge O, Mihara K (2016). Drp1-dependent mitochondrial fission via MiD49/51 is essential for apoptotic cristae remodeling. J Cell Biol.

[51] Smirnova E, Griparic L, Shurland DL, van der Bliek AM (2001). Dynamin-related protein Drp1 is required for mitochondrial division in mammalian cells. Mol Biol Cell.

[52] Parone PA (2006). Inhibiting the mitochondrial fission machinery does not prevent Bax/Bak-dependent apoptosis. Mol Cell Biol.

[53] Wang P (2015). Dynamin-related protein Drp1 is required for Bax translocation to mitochondria in response to irradiation-induced apoptosis. Oncotarget.

[54] Grosse L (2016). Bax assembles into large ring-like structures remodeling the mitochondrial outer membrane in apoptosis. Embo J.

[55] Estaquier J, Arnoult D (2007). Inhibiting Drp1-mediated mitochondrial fission selectively prevents the release of cytochrome c during apoptosis. Cell Death Differ.

[56] Ugarte-Uribe Begoña, García-Sáez Ana J. (2017). Apoptotic foci at mitochondria: in and around Bax pores. Philosophical Transactions of the Royal Society B: Biological Sciences.

[57] Salvador-Gallego R (2016). Bax assembly into rings and arcs in apoptotic mitochondria is linked to membrane pores. Embo J.

[58] Kuwana T, Olson NH, Kiosses WB, Peters B, Newmeyer DD (2016). Pro-apoptotic Bax molecules densely populate the edges of membrane pores. Sci Rep.

[59] Chanez AL, Hehl AB, Engstler M, Schneider A (2006). Ablation of the single dynamin of T. brucei blocks mitochondrial fission and endocytosis and leads to a precise cytokinesis arrest. J Cell Sci.

[60] Yoon Y, Pitts KR, McNiven MA (2001). Mammalian dynamin-like protein DLP1 tubulates membranes. Mol Biol Cell.

[61] Antonny B (2016). Membrane fission by dynamin: what we know and what we need to know. Embo j.

[62] Bardai FH, D’Mello SR (2011). Selective toxicity by HDAC3 in neurons: regulation by Akt and GSK3beta. J Neurosci.

[63] Van Bergen NJ (2009). Recharacterization of the RGC-5 retinal ganglion cell line. Invest Ophthalmol Vis Sci.

[64] Krishnamoorthy RR, Clark AF, Daudt D, Vishwanatha JK, Yorio T (2013). A forensic path to RGC-5 cell line identification: lessons learned. Invest Ophthalmol Vis Sci.

[65] Semaan SJ, Li Y, Nickells RW (2010). A single nucleotide polymorphism in the Bax gene promoter affects transcription and influences retinal ganglion cell death. ASN neuro.

[66] Semaan SJ, Nickells RW (2010). The apoptotic response in HCT116BAX^−/−^ cancer cells becomes rapidly saturated with increasing expression of a GFP-BAX fusion protein. BMC Cancer.

[67] Goldstein JC, Waterhouse NJ, Juin P, Evan GI, Green DR (2000). The coordinate release of cytochrome c during apoptosis is rapid, complete and kinetically invariant. Nat Cell Biol.

[68] Friedman JR (2011). ER tubules mark sites of mitochondrial division. Science.

[69] Kim KY (2015). DRP1 inhibition rescues retinal ganglion cells and their axons by preserving mitochondrial integrity in a mouse model of glaucoma. Cell Death Dis.

