# Supplementary material for: Completion of BAX recruitment correlates with mitochondrial fission during apoptosis
Source: Sci Rep. 2019 Nov 12;9:16565. doi: 10.1038/s41598-019-53049-w (PMC6851089; doi:10.1038/s41598-019-53049-w)
Supplement: Supplementary file 2 — Supplemental Info [file 41598_2019_53049_MOESM2_ESM.pdf]

Supplemental Material

Completion of BAX recruitment correlates with mitochondrial fission during apoptosis

Maes, ME[1,2], Grosser, JA[1], Fehrman, RL[1], Schlamp, CL[1,3], Nickells, RW[1]*.

[1]Department of Ophthalmology and Visual Sciences, University of Wisconsin – Madison, Madison, WI, USA
[2]Institute of Science and Technology Austria, Klosterneuburg, Austria
[3]Deceased

SUMMARY OF SUPPLEMENTAL MATERIAL

Supplemental Video 1. Time lapse live-cell imaging of BAX recruitment and mitochondrial volume changes during apoptosis

Supplemental Figure 1. Stages of BAX recruitment.

Supplemental Figure 2. Mitochondrial volume changes as a function of BAX recruitment in ARPE-19 cells treated with staurosporine.

Supplemental Figure 3. Mitochondrial morphology changes in HCT116$^{BAX-/-/BAK-/-}$ cells expressing wild type and P168A BAX constructs.

Supplemental Figure 4. Mitochondrial organelle axial length measurements in HCT116$^{BAX-/-/BAK-/-}$ cells expressing wild type and P168A BAX constructs.

Supplemental Figure 5. Cytochrome c-GFP release in HCT116$^{BAX-/-/BAK-/-}$ cells expressing wild type and P168A BAX constructs.

Supplemental Figure 6. Endogenous BAX activation profile in wild type and *DRP1*$^{-/-}$ HeLa cells treated with staurosporine.

Supplemental Figure 7. Summary of BAX recruitment curves.

Supplemental Video 1. Time lapse live-cell imaging of BAX recruitment and mitochondrial volume changes during apoptosis. A movie of a differentiated 661W cell undergoing apoptosis after the forced expression of exogenous Histone Deacetylase 3 (HDAC3). The cell was previously nucleofected with plasmids expressing GFP-BAX and mito-BFP. After 24 hrs, the cell was transfected with a plasmid encoding HDAC3. (a) GFP-BAX becomes recruited to mitochondrial foci, moving from a diffuse cytosolic localization to a punctate pattern. (b) The mito-BFP labeling was analyzed in 20-25 optical sections using IMARIS. Contiguous organelles were identified and their volume was measured and represented as a heat map. Both images are synchronized to the same time frame.

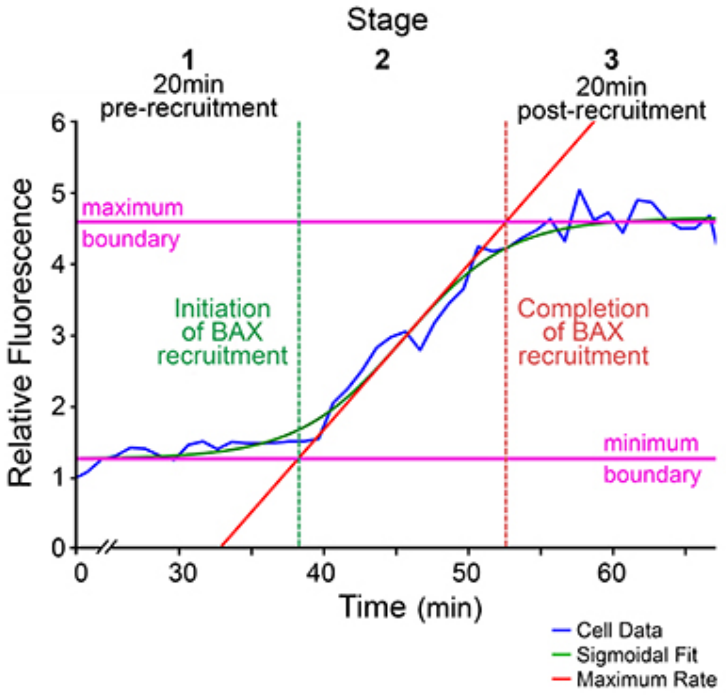

Supplemental Figure 1. Stages of BAX recruitment.
A representative recruitment curve of a fluorescent BAX fusion protein at a single mitochondrial focus is shown (see Figure 1A for representative time lapse images). Intensity measurements are normalized to one. As BAX is recruited to the mitochondrion, fluorescence intensity increases, and follows a sigmoid shaped pattern. Raw data are fit with sigmoid curves. The initiation (green dashed line) and completion (red dashed line) of recruitment are calculated by determining the x-values at which the slope of the line at the inflection point crosses the minimum and maximum of the curve. Stage 1, 2, and 3, are the three portions of the curve carved out by the initiation and completion lines, as indicated on the graph.

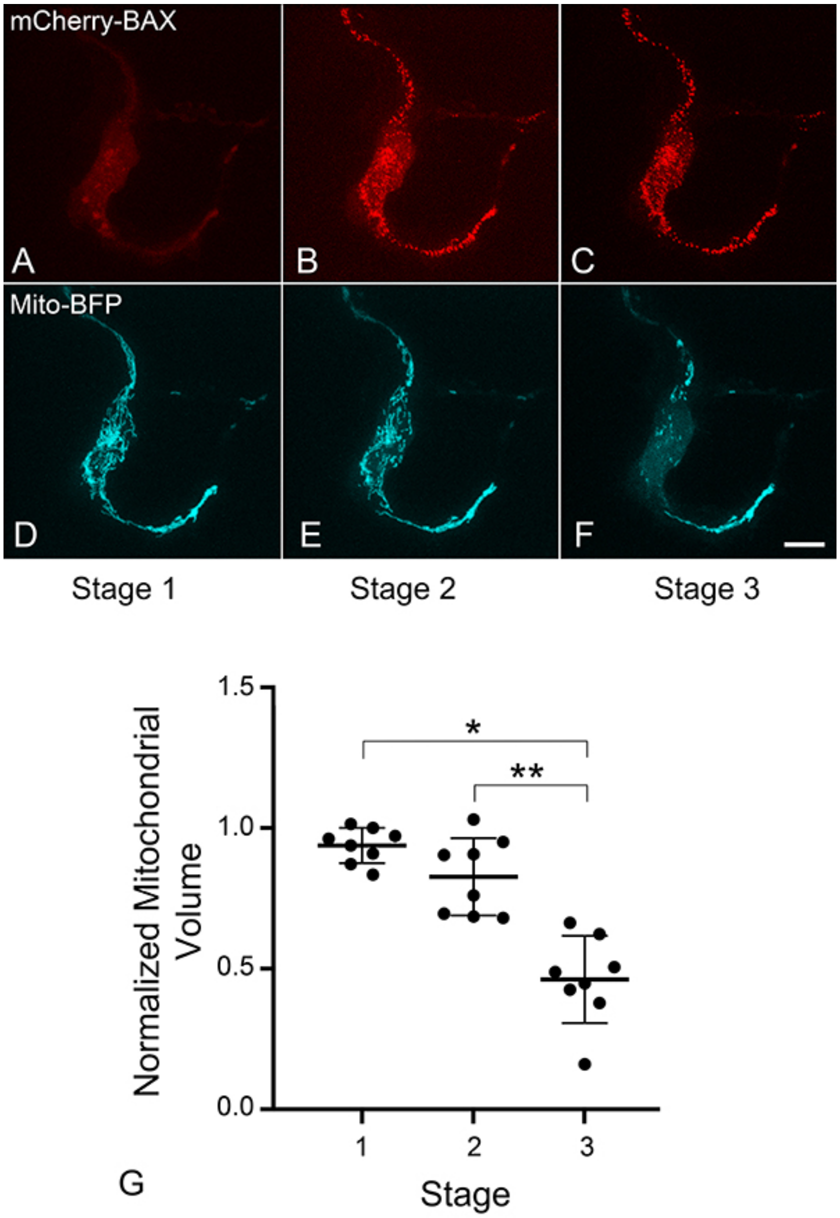

Supplemental Figure 2. Mitochondrial volume changes as a function of BAX recruitment in ARPE-19 cells treated with staurosporine.

(A-F) Images from a time-lapse recording of a single cell during the process of mCherry-BAX recruitment (A-C) induced by staurosporine treatment, and (D-E) the corresponding images of mito-BFP labeled mitochondria. The images are representative of a 10 minute interval prior to the initiation of BAX recruitment (A, D – Stage 1), during recruitment (B, E – Stage 2), and up to 30 minutes after completion of recruitment (C, F – Stage 3). Size bar = 5 µm. (G) Graph showing the relative average mitochondrial volume (normalized to 1.0 for each cell at 10 minutes prior to BAX recruitment, in Stage 1) of n = 8 different cells in each stage. There is no statistical difference between mitochondrial volume between cells in Stages 1 and 2 ($p = 0.06$), but volumes are significantly smaller in Stage 3 cells (*$p < 0.001$ compared to Stage 1 and **$p < 0.001$ compared to Stage 2).

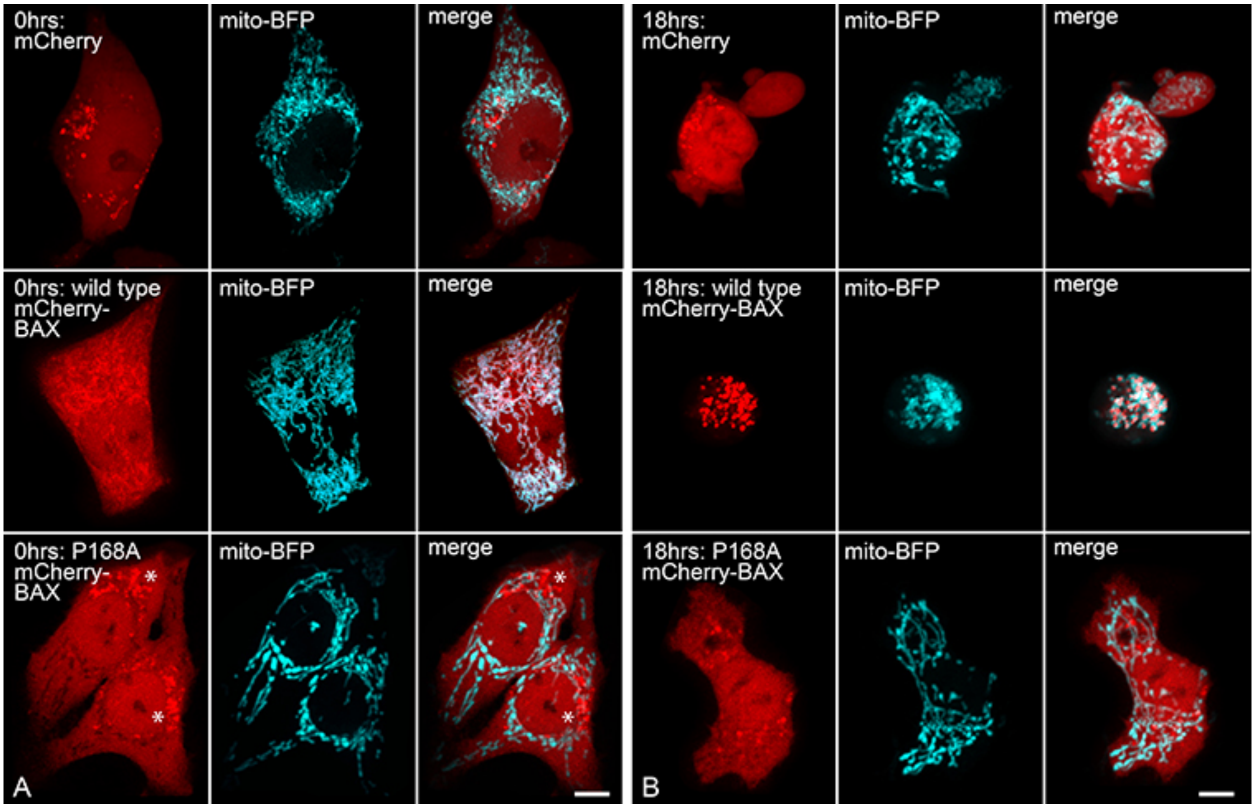

Supplemental Figure 3. Mitochondrial morphology changes in HCT116$^{\textit{BAX-/-/BAK-/-}}$ cells expressing wild type and P168A BAX constructs.

Mito-BFP was used to monitor mitochondrial morphology (pseudo-colored teal). (A) mCherry, wild type mCherry-BAX, or P168A mCherry-BAX were overexpressed for three days to allow ample time for mitochondria morphology recovery. Note that after this period, some of the reporter proteins formed aggregates (asterisks) that did not correspond to mitochondria. We interpret these aggregates as excess protein that have become incorporated into lysosomes. 0hrs is considered the baseline recovery morphology, prior to staurosporine (STS) addition. Mitochondria in cells expressing empty mCherry are small and fragmented, as typically seen in HCT116$^{BAX-/-/BAK-/-}$ cells [25,41]. Both wild type and P168A mCherry-BAX expressing cells exhibited more elongated, filamentous mitochondrial networks after three days of expression. (B) 18hrs after apoptotic induction using 1 μM STS, cells lacking BAX (mCherry only) still had small, fragmented mitochondria. Cells expressing wild type mCherry-BAX exhibited BAX recruitment to the mitochondria, and mitochondrial fragmentation. Conversely, P168A mCherry-BAX remained cytoplasmic and the cells did not show mitochondrial fragmentation after STS addition. Size bars = 5 μm.

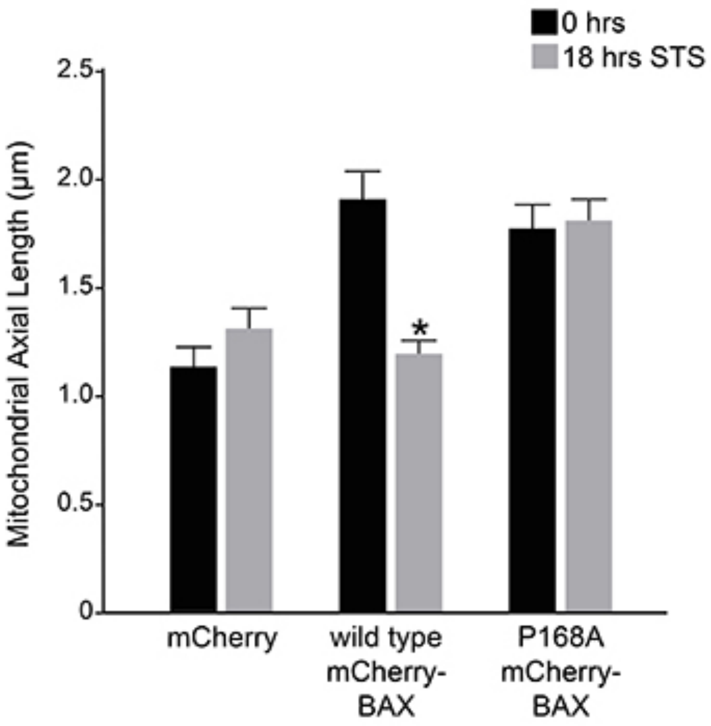

Supplemental Figure 4. Mitochondrial organelle axial length measurements in HCT116$^{BAX-/-/BAK-/-}$ cells expressing wild type and P168A BAX constructs.
To corroborate the volume measurement data described in Figure 2, we also measured the longest axial length of organelles identified in 3-dimensional renderings of HCT116$^{BAX-/-/BAK-/-}$ cells expressing wild type and P168A BAX constructs. Cells expressing mCherry alone have significantly shorter mitochondria than cells expressing either wild type mCherry-BAX, or the P168A mCherry-BAX mutant ($p < 0.0001$, comparing groups at 0 hrs). After addition of 1 µM staurosporine (STS), only cells expressing wild type mCherry-BAX exhibit a decrease in mitochondrial length (*$p = 2.6 \times 10^{-5}$).

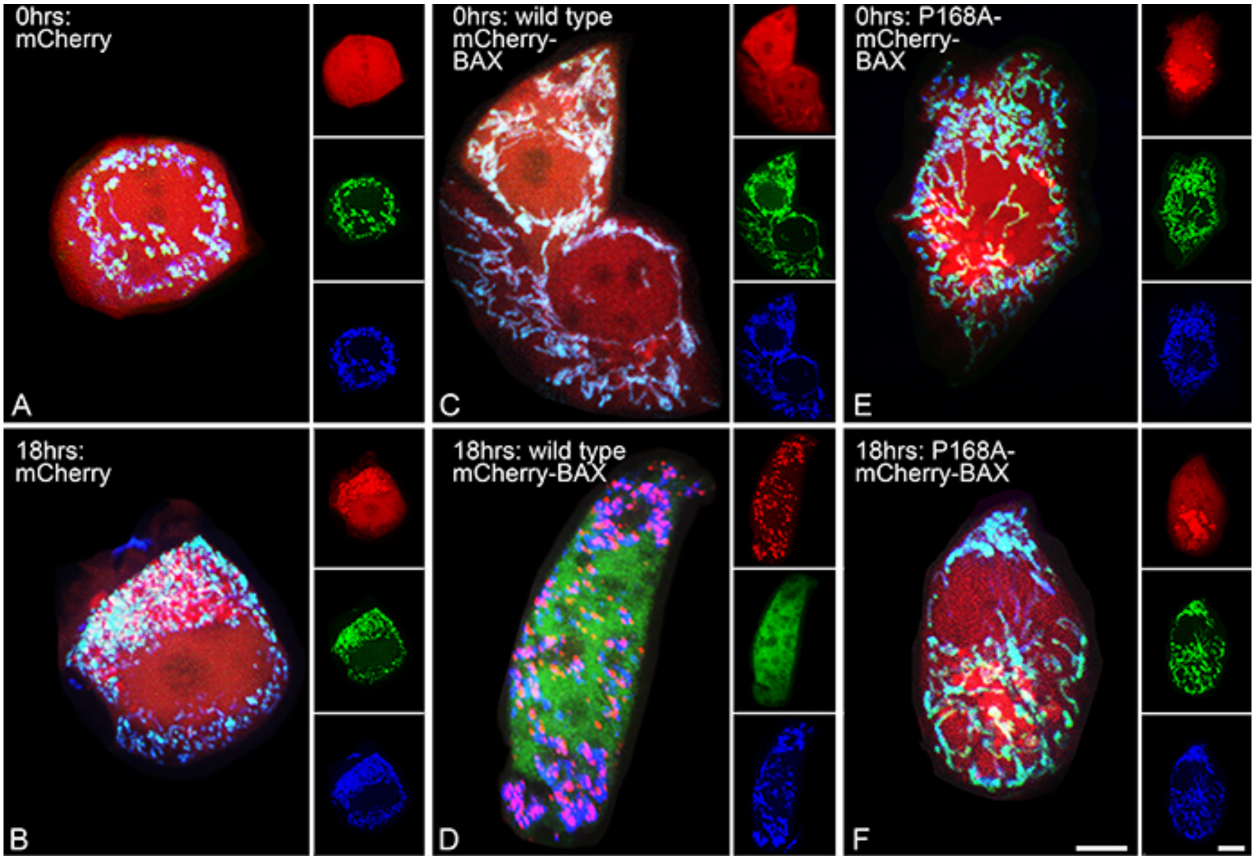

Supplemental Figure 5. Cytochrome c-GFP release in HCT116$^{BAX-/-/BAK-/-}$ cells expressing wild type and P168A BAX constructs.
HCT116$^{BAX-/-/BAK-/-}$ cells expressing cytochrome c-GFP, mito-BFP and either mCherry (A, B), wild type mCherry-BAX (C, D), or P168A mCherry-BAX (E, F) were challenged with 1 µM staurosporine (STS). All conditions showed cytochrome c-GFP localization at the mitochondria at baseline (A, C, E), but only mCherry (B) and P168A mCherry-BAX (F) maintained that localization after STS. (D) Wild type mCherry-BAX recruited to the mitochondria after STS, leading to cytochrome c release and cytoplasmic localization. The insets for each panel show individual channels for each reporter protein, while the full-sized panel is the merged image. Size bars = 5 µm.

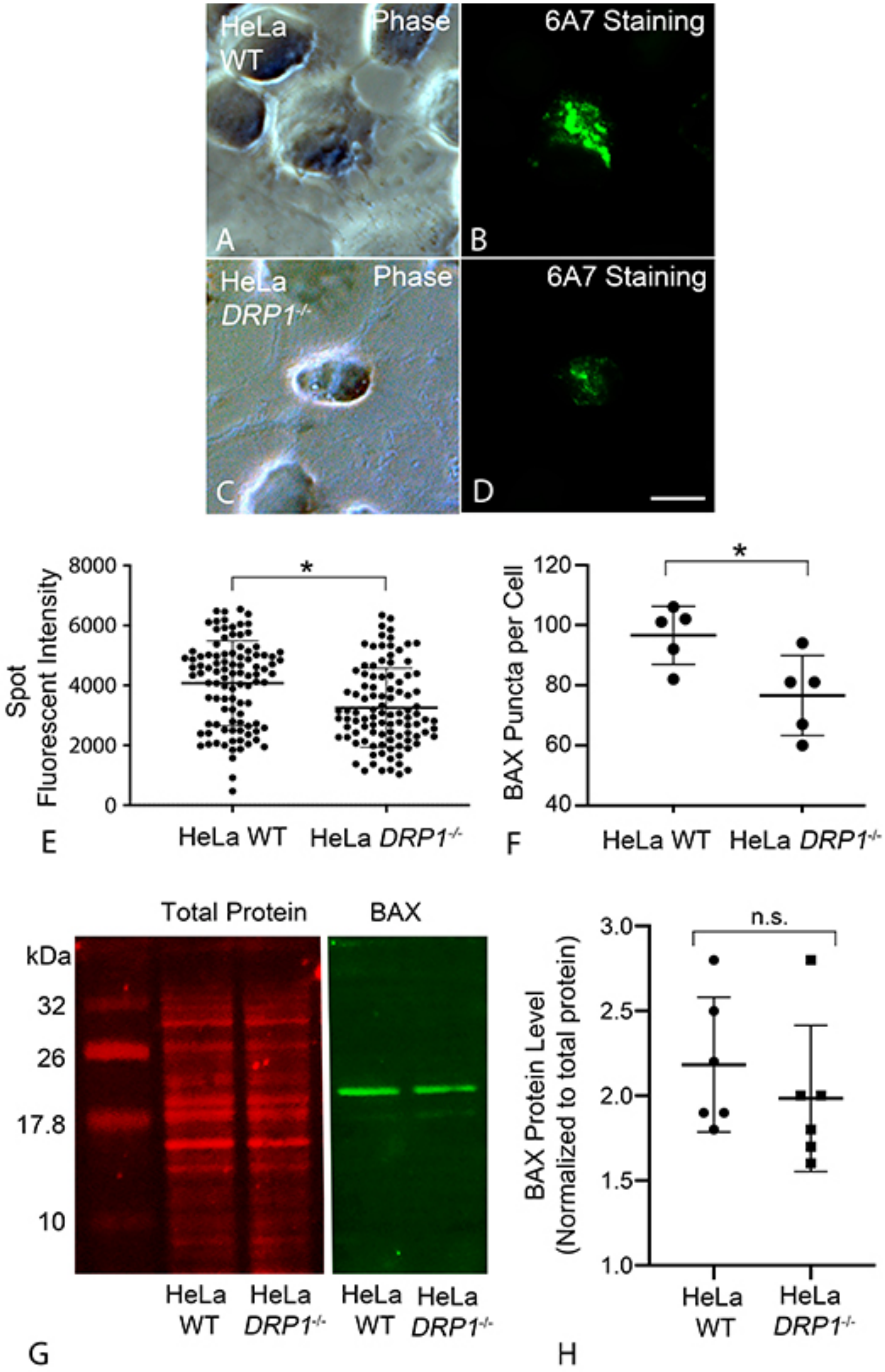
HeLa WT
Phase
6A7 Staining
A
B
HeLa DRP1-/-
Phase
6A7 Staining
C
D
Spot Fluorescent Intensity
8000
6000
4000
2000
0
*
HeLa WT
HeLa DRP1-/-
E
BAX Puncta per Cell
120
100
80
60
40
*
HeLa WT
HeLa DRP1-/-
F
Total Protein
BAX
kDa
32
26
17.8
10
HeLa WT
HeLa DRP1-/-
HeLa WT
HeLa DRP1-/-
G
BAX Protein Level (Normalized to total protein)
3.0
2.5
2.0
1.5
1.0
n.s.
HeLa WT
HeLa DRP1-/-
H

Supplemental Figure 6. Endogenous BAX activation profile in both wild type and *DRP1*$^{-/-}$ HeLa cells treated with staurosporine.

(A, B) Wild type (WT) and (C, D) *DRP1*$^{-/-}$ HeLa cells were challenged with 1 μM staurosporine for 3 hours, after which they were fixed and stained for activated BAX using the 6A7 monoclonal antibody. Size bar = 7 μm. Both genotypes exhibited positive staining for activated BAX, although staining intensity was generally reduced in *DRP1*$^{-/-}$ cells (immuno-fluorescent images taken at fixed laser intensity and exposure times). To assess this, fluorescent intensity was measured in individual spots in 5 positive cells from each group (minimum of 20 puncta per cell) and plotted as a scatter plot (E). Mean (± SD) spot intensity in WT cells was 4073.9 ± 1417.2 pixels/punctum compared to 3258.5 ± 1319.6 pixels/punctum for DRP1-deficient cells, representing a 25.6% decrease in staining intensity (*p = 3.8 x 10^{-5}). (F) The number of puncta per cell was also measured in the same 5 cells in each group. Wild type HeLa cells yielded 96.9 ± 9.6 puncta per cell, while *DRP1*$^{-/-}$ cells exhibited significantly fewer puncta (70.6 ± 9.8, *p = 0.003). (G) Measurement of BAX protein levels in WT and *DRP1*$^{-/-}$ HeLa cells. Representative LI-COR scanned image of total protein loading (700 nm) and of BAX immunostaining (800 nm) on a transfer membrane of cell extracts separated on a 12% polyacrylamide SDS gel. (H) Graphical representation of BAX levels normalized to total protein levels. The data shown is of 2 separate cell preparations of each genotype, and BAX was measured in 3 different protein concentrations for each sample. No significant difference (n.s.) was detected in BAX levels between the different genotypes of HeLa cells (p = 0.423).

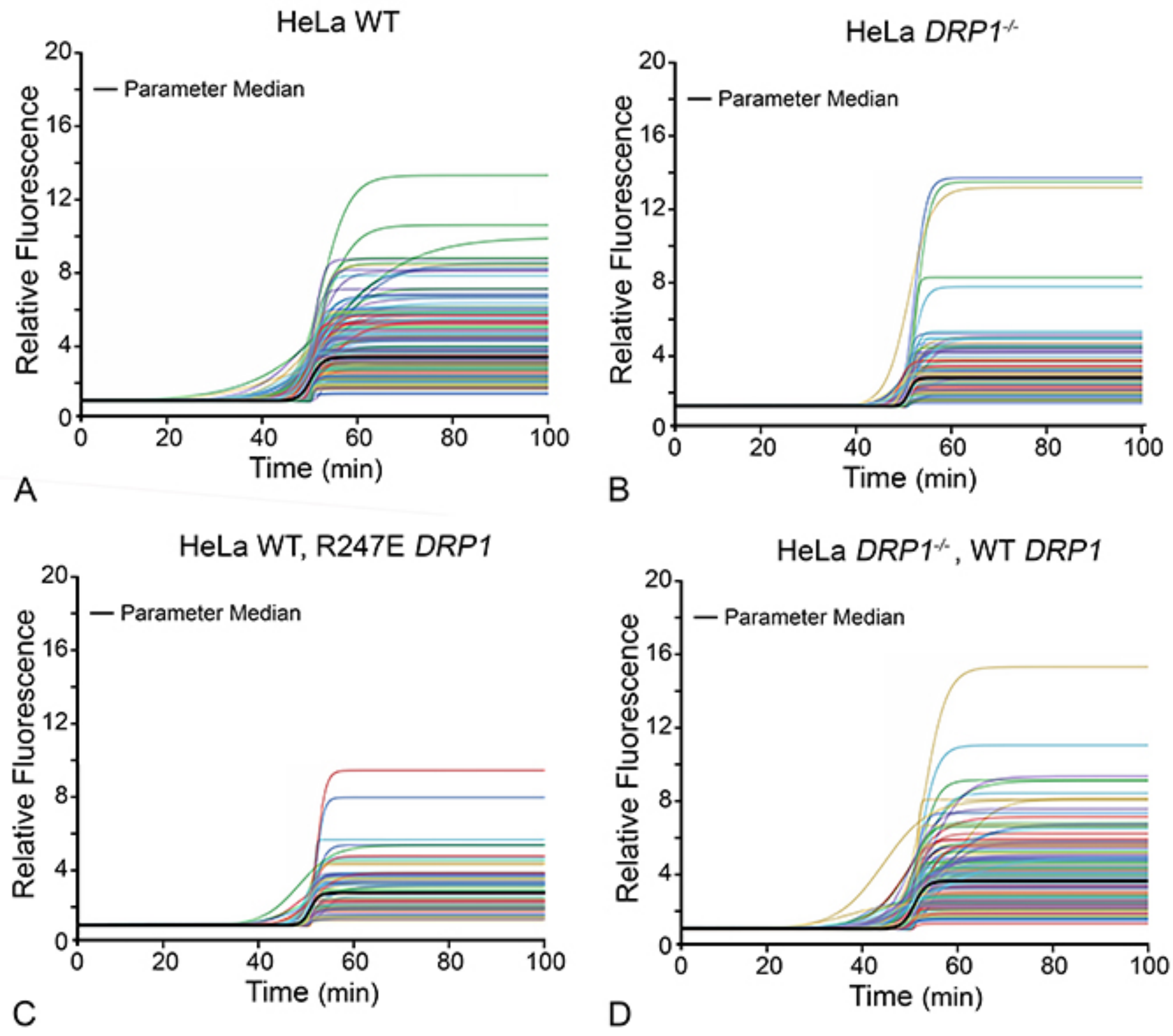

Supplemental Figure 7. Summary of BAX recruitment curves.
The population of BAX recruitment curves are plotted for (A) HeLa wild type (WT), (B) HeLa$^{DRP1-/-}$, (C) HeLa WT + R247E DRP1, and (D) HeLa$^{DRP1-/-}$ + WT DRP1 with a representative curve (black) which is the median of all sigmoid curves when aligned at their inflection point (n = 156, 87, 59, 129 respectively).
